# A group matrix representation relevant to scales of measurement of clinical disease states via stratified vectors

**DOI:** 10.1186/s12976-016-0031-8

**Published:** 2016-02-09

**Authors:** Jitsuki Sawamura, Shigeru Morishita, Jun Ishigooka

**Affiliations:** Department of Psychiatry, Tokyo Women’s Medical University, Tokyo, Japan; Depression Prevention Medical Center, Inariyama Takeda Hospital, Kyoto, Japan

**Keywords:** Group, Ring, Operation, Stratified vector, Matrix representation, Clinical medicine

## Abstract

**Background:**

Previously, we applied basic group theory and related concepts to scales of measurement of clinical disease states and clinical findings (including laboratory data). To gain a more concrete comprehension, we here apply the concept of matrix representation, which was not explicitly exploited in our previous work.

**Methods:**

Starting with a set of orthonormal vectors, called the basis, an operator R_j_ (an N-tuple patient disease state at the j-th session) was expressed as a set of stratified vectors representing plural operations on individual components, so as to satisfy the group matrix representation.

**Results:**

The stratified vectors containing individual unit operations were combined into one-dimensional square matrices [R_j_]s. The [R_j_]s meet the matrix representation of a group (ring) as a K-algebra. Using the same-sized matrix of stratified vectors, we can also express changes in the plural set of [R_j_]s. The method is demonstrated on simple examples.

**Conclusions:**

Despite the incompleteness of our model, the group matrix representation of stratified vectors offers a formal mathematical approach to clinical medicine, aligning it with other branches of natural science.

## Background

For much of the 20th century, abstract algebras such as groups and related structures, including rings, group rings, and fields, have been adopted in various fields of natural science [1–5]. Symmetry treatments of dynamic phenomena have been integral to simplifying, unifying, and integrating formal representations of the natural sciences, such as chemistry [6], physics [7–10], molecular biology [11–15], and anthropology [16].

Unfortunately, clinical medicine has not attained a similar level of sophistication that would link it to the natural sciences. In 1946, Stevens proposed four classifications of measurements: “Nominal”, “Ordinal”, “Interval” and “Ratio”. These categories have become widely used in the medical fields and have played important roles in constructing and interpreting scales [17]. Previously, we applied basic group theory and related concepts to measurement scales abstracted from clinical disease states and clinical findings, including laboratory data, using a vector product approach. We reformulated the Stevens classification in an abstract algebra-like scheme; namely, we replaced Stevens’ ordinal scale with ‘Abelian modulo additive group’ plus ‘zero’, the interval scale with ‘Abelian additive group’ and the ratio scale with ‘field’. Furthermore, various schemes describing the assessment of patient states were organized into vectors [18, 19]. This vector-like notation, which adopts a hierarchical-cluster form, enables sophisticated data-mining and dataset combinations.

Previously, we defined operations using modulo arithmetic and simple arithmetic. The respective symptoms were assessed by their severity based on a p-grading (where p denotes a prime number). The modulo-p arithmetic (addition, subtraction, multiplication and division) was collectively denoted by ‘†’. Theoretical or related group products were expressed as r_(j)ν_ † r_(j→k)ν_ (mod p) = r_(k)ν_ (mod p), denoting that r_(j)ν_ (mod p) is the *v*-th component of R_j_ (severity of the *v*-th symptom or laboratory data in p-graded form). Similarly, r_(j→k)ν_ (mod p) is the *v*-th component of R_(j→k)_ (an operator that changes the severity of the *v*-th symptom). Combining these expressions, we obtain r_(k)ν_ (mod p), denoting the *v*-th component of R_k_ (a result of changing the *v*-th symptom). The set r_(j)ν_ comprises Z_p_ = {1, 2,…, p −1}. A change of a global state is expressed as R_j_ (mod p) † R_(j→k)_ (mod p) = {R_j_ † R_(j→k)_} (mod p) = {R_j_ † (R_(j→0)_ † R_(0→k)_)} (mod p) = {R_j_ † R_j_^-1^ † R_k_} (mod p) = {(R_j_ † R_j_^-1^) † R_k_} (mod p) = {R_0_ † R_(0→k)_} (mod p) = R_k_ (mod p) (e.g., *p* = 7), where j, k = 1, 2, 3,… are positive integers, R_j_ expresses a patient’s disease state as a combination of the respective symptom severities, and R_(j→k)_ is an operator that changes the disease state to R_k_ by algebraically acting on R_j_. This global state change can be described as a Cartesian vector Z_p_^×N^ with components *v* from 1 to N. For a more detailed interpretation, we denote ‘arithmetic’ by ‘#’, ‘ordinal addition’ by ‘°’, ‘modulo p (prime) arithmetic by ‘†’, ‘modulo addition’ by ‘*’, ‘non-modulo/modulo p multiplication’ by ‘×’, and ‘non-modulo/modulo p division’ by ‘/’. Collectively, these symbols express non-modulo or modulo p arithmetic operations, which were confirmed to satisfy the postulates of groups, rings or fields [18, 19]. Replacing ‘†’ with ‘#’, which collectively denotes non-modular ordinal arithmetic, we can write ‘R_j_ # R_(j→k)_ = R_k_’, which satisfies similar conditions. Additionally, we can collectively denote ‘†’ and ‘#’ by ‘◊’. In this notation, a series of disease states R_1_, R_2_, R_3_,…, R_m-1_, R_m_ can be expressed as a combination of operators

1 $$ {\mathrm{R}}_1\lozenge {{\mathrm{R}}_{\Big(1}}_{\to 2\Big)}\lozenge {{\mathrm{R}}_{\Big(2}}_{\to 3\Big)}\lozenge \dots \lozenge {{\mathrm{R}}_{\Big(\mathrm{m}-2}}_{\to \mathrm{m}-1\Big)}\lozenge {{\mathrm{R}}_{\Big(\mathrm{m}-1}}_{\to \mathrm{m}\Big)} = {\mathrm{R}}_{\mathrm{m}}. $$

The equivalent notation for the *v*-th component is

2 $$ {{\mathrm{r}}_{\Big(1}}_{\Big)\upnu}\lozenge {{\mathrm{r}}_{\Big(1}}_{\to 2\Big)\upnu}\lozenge {{\mathrm{r}}_{\Big(2}}_{\to 3\Big)\upnu}\lozenge \dots \lozenge {{\mathrm{r}}_{\Big(\mathrm{m}-2}}_{\to \mathrm{m}-1\Big)\upnu}\lozenge {{\mathrm{r}}_{\Big(\mathrm{m}-1}}_{\to \mathrm{m}\Big)\upnu}={\mathrm{r}}_{\left(\mathrm{m}\right)\upnu},\left(\mathrm{where}\kern0.5em v = 1,2,\dots, \mathrm{N}\right). $$

In the following description, we adopt the most general symbol ‘◊’ (meaning ‘†’ and/or ‘#’), which is sufficient to express our ideas.

To simplify the discussion, we also regard non-modulo/modulo-p division as a multiplication operator; modulo-p division is treated as 1/R_j_ (mod p) = R_j_^−1^ (mod p), where R_j_^−1^ satisfies gcd (R_j_, R_j_^−1^) = 1 (mod p) (gcd: greatest common divisor); that is, r_(j)ν_ × r_(j)ν_^−1^ = 1 (mod p) holds for each component *v* of R_j_. Moreover, a division by r_(j)ν_ (where r_(j)ν_ is an integer number in modulo division, and a complex number in non-modulo division) in R_j_ is considered as a multiplication by 1/r_(j)ν_; consequently, division by R_j_ is a multiplication by 1/R_j_. For this reason, it is sufficient that ◊ constitutes addition or multiplication (non-modulo/modulo) alone.

In our previous report [19], we provided no explicit matrix representations. Group representations are mappings from an abstract group to matrices based on linear operators acting on vector spaces. Group operations and direct products of groups correspond to matrix multiplications and tensor products, respectively [9, 20–22]. Moreover, in our model, operations on individual components of R_j_ are mixtures of modulo/non-modulo arithmetic; for example,

3 $$ \begin{array}{l}{\mathrm{R}}_{\mathrm{j}} = \left[{\mathrm{r}}_{\left(\mathrm{j}\right)1}\left(\mathrm{modulo}\ {\mathrm{p}}_1\kern0.5em \mathrm{addition}\right)\ \left|\ {\mathrm{r}}_{\left(\mathrm{j}\right)2}\left(\mathrm{modulo}\ {\mathrm{p}}_2\kern0.5em \mathrm{multiplication}\right)\ \right|\ {\mathrm{r}}_{\left(\mathrm{j}\right)3}\left(\mathrm{n}\mathrm{o}\mathrm{n}\hbox{-} \mathrm{modulo}\ \mathrm{addition}\right)\ \right|\dots \Big|\\ {}{\mathrm{r}}_{\left(\mathrm{j}\right)\upnu}\left(\mathrm{n}\mathrm{o}\mathrm{n}\hbox{-} \mathrm{modulo}\ \mathrm{division}\right)\ \left|\dots \right|\ {\mathrm{r}}_{\left(\mathrm{j}\right)\mathrm{N}}\left(\mathrm{n}\mathrm{o}\mathrm{n}\hbox{-} \mathrm{modulo}\ \mathrm{arithmetic}\right)\Big]\ \left(v = 1,2,\dots, \mathrm{N}\right).\end{array} $$

In this representation, an ordinal matrix treatment is difficult or impossible, because the plural rules governing the different rows/columns in a square matrix are expressed as plural modulo-p_1_/p_2_ arithmetic or non-modulo arithmetic. This notation seems to violate the consistency requirement for sums of matrix elements, because different operations acting on the matrix components yield all combinations of these operations in the matrix product, such as ‘r_(j)ν_ = a·r_(j)1_ (modulo p_1_ addition) + b·r_(j)2_ (modulo p_2_ multiplication) + c·r_(j)3_ (non-modulo addition)… + d·r_(j)ν_ (non-modulo division)… + e·r_(j)N_ (non-modulo arithmetic) (where a, b, c, d and e are appropriate numbers) (#). If plural operational rules are mixed in a unique matrix, equation (#) will become nonsensical. Conventionally, an operational rule for respective elements should be uniquely expressed (as a unique operational unit); for example, A (mod p_1_), where A is a matrix. Hence, we require a method that individually treats each component with no interactions between respective components. A mixture of r_(j)ν_ and r_(j)μ_ (*v* ≠ *μ*) in the same element is undesirable. In this sense, our construct differs from ordinal matrices covering *n*-dimensional space. In our methodology, respective symptom/clinical data are independently treated within individual components. Interactions should be limited to the same components at different session numbers *j*. To satisfy these conditions and explore the formal ranges of our model, especially, to find a matrix representation of the vector-like notation (3) with mixed operations [19], we attempt to describe the R_j_s in a novel form, while preserving (as far as possible) the conventional group matrix representation. In the following discussion, the ‘perpendicular’ expression of vectors (matrices) and bases is unique to the present article. For clarification, the method is demonstrated in a simple example.

## Methods

### Model assumptions

#### §1. A composition of stratified vectors that separately expresses plural operations in individual components

To visualize its behaviors, a group is often described as a linear combination of all matrix elements. Fundamentally, the matrix representation depends upon the basis and operations. The matrix representation provides a tangible view of the specific characteristics of groups and the action of operators on the basis. Here, we add an optional definition. Let R_j_ ∈ M be a complex variable (or complex number) containing N-tuple components of (3) (where N is a natural number). The N-tuple product R_j_ is expressed as a stratified vector in set M. In this regard, M is not merely equal to C^×N^’ because R_j_ is constructed from operations such as Z_p1_ (= {0, 1, 2,…, p_1_ − 1}) × Z_p2_ (= {0, 1, 2,…, p_2_ − 1}) × C_3_ (complex number) × C_4_ × … × C_N_, in which each component can have different operations and/or units. Instead, we write M ≡ {Z_p_ or C ^×N^, ◊}. To express a matrix of arbitrary R_j_s while avoiding the inconsistency among different components with different operational rules, we rewrite the *v*-th layer-component of R_j_ as r_(j)ν_ (= ‹R_j_›_ν_). We refer to this novel form as a *perpendicularly stratified vector* R_j_ (meaning a column vector that is perpendicular to the plane of the paper, not a column vector on the page. However, as such a vector cannot be portrayed on the page, it is written as a standard column vector). The components of R_j_ are perpendicularly arrayed and ordered from bottom to top. For easy visualization, the R_j_s are displayed as column vectors with their components in descending order from top to bottom:

4 $$ {\mathrm{R}}_{\mathrm{j}}=\left[\begin{array}{c}\hfill \mathrm{r}\left(\mathrm{j}\right)1\kern1.5em \left( \mod \kern0.5em \mathrm{p}1\right)\hfill \\ {}\hfill \mathrm{r}\left(\mathrm{j}\right)2\kern1.5em \left( \mod \kern0.5em \mathrm{p}2\right)\hfill \\ {}\hfill \mathrm{r}\left(\mathrm{j}\right)3\kern0.5em \hfill \\ {}\hfill \vdots \kern0.5em \hfill \\ {}\hfill \mathrm{r}\left(\mathrm{j}\right)\upnu \kern0.5em \hfill \\ {}\hfill \vdots \hfill \\ {}\hfill \mathrm{r}\left(\mathrm{j}\right)\mathrm{N}\hfill \end{array}\right]\kern0.5em ,\ {\mathrm{R}}_{\left(\mathrm{j}\to \mathrm{k}\right)}=\left[\begin{array}{c}\hfill \mathrm{r}\left(\mathrm{j}\to \mathrm{k}\right)1\kern1.5em \left( \mod \kern0.5em \mathrm{p}1\right)\hfill \\ {}\hfill \mathrm{r}\left(\mathrm{j}\to \mathrm{k}\right)2\kern1.5em \left( \mod \kern0.5em \mathrm{p}2\right)\hfill \\ {}\hfill \mathrm{r}\left(\mathrm{j}\to \mathrm{k}\right)3\kern0.5em \hfill \\ {}\hfill \vdots \kern0.5em \hfill \\ {}\hfill \mathrm{r}\left(\mathrm{j}\to \mathrm{k}\right)\upnu \kern0.5em \hfill \\ {}\hfill \vdots \hfill \\ {}\hfill \mathrm{r}\left(\mathrm{j}\to \mathrm{k}\right)\mathrm{N}\hfill \end{array}\right]\kern0.5em ,\kern0.5em {\mathrm{R}}_{\mathrm{j}}\lozenge {\mathrm{R}}_{\left(\mathrm{j}\to \mathrm{k}\right)}=\left[\begin{array}{c}\hfill \mathrm{r}\left(\mathrm{j}\right)1\lozenge \mathrm{r}\left(\mathrm{j}\to \mathrm{k}\right)1\kern1.5em \left( \mod \kern0.5em \mathrm{p}1\right)\hfill \\ {}\hfill \mathrm{r}\left(\mathrm{j}\right)2\lozenge \mathrm{r}\left(\mathrm{j}\to \mathrm{k}\right)2\kern1.5em \left( \mod \kern0.5em \mathrm{p}2\right)\hfill \\ {}\hfill \mathrm{r}\left(\mathrm{j}\right)3\lozenge \mathrm{r}\left(\mathrm{j}\to \mathrm{k}\right)3\kern0.5em \hfill \\ {}\hfill \vdots \kern0.5em \hfill \\ {}\hfill \mathrm{r}\left(\mathrm{j}\right)\nu \lozenge \mathrm{r}\left(\mathrm{j}\to \mathrm{k}\right)\upnu \kern0.5em \hfill \\ {}\hfill \vdots \hfill \\ {}\hfill \mathrm{r}\left(\mathrm{j}\right)\mathrm{N}\lozenge \mathrm{r}\left(\mathrm{j}\to \mathrm{k}\right)\mathrm{N}\hfill \end{array}\right]\kern0.5em =\left[\begin{array}{c}\hfill \mathrm{r}\left(\mathrm{k}\right)1\kern1.5em \left( \mod \kern0.5em \mathrm{p}1\right)\hfill \\ {}\hfill \mathrm{r}\left(\mathrm{k}\right)2\kern1.5em \left( \mod \kern0.5em \mathrm{p}2\right)\hfill \\ {}\hfill \mathrm{r}\left(\mathrm{k}\right)3\kern0.5em \hfill \\ {}\hfill \vdots \kern0.5em \hfill \\ {}\hfill \mathrm{r}\left(\mathrm{k}\right)\upnu \kern0.5em \hfill \\ {}\hfill \vdots \hfill \\ {}\hfill \mathrm{r}\left(\mathrm{k}\right)\mathrm{N}\hfill \end{array}\right]\kern0.5em  = {\mathrm{R}}_{\mathrm{l}}\left(\in \mathrm{M}\right). $$

In (4), which is presented for illustrative purposes only, the operations in the *v*-th layer (component) are arbitrarily assigned, and the components of the ‘virtual column vectors’ are placed in descending order, where the *v*-th component from the bottom denotes

5 $$ \mbox{\fontencoding{T1}\fontfamily{cmr}\selectfont\char"0E} {\mathrm{R}}_{\mathrm{j}}{\mbox{\fontencoding{T1}\fontfamily{cmr}\selectfont\char"0F}}_{\upnu} = {\mathrm{r}}_{\left(\mathrm{j}\right)\upnu}\left(\upnu = 1,2,\dots \mathrm{N}\right). $$

In this way, all elements R_j_ (∈M) are interpretable as *perpendicularly stratified* vectors. We can also regard R_j_ as a one-dimensional square matrix [R_j_] composed of N-tuple stratified components r_(j)ν_ (where *v* = 1,2,…N). Also, each vector e_(ν)_ can be viewed as a 1 × 1 square matrix [e_(ν)_] where e_(ν)_ is a unit basis vector composed of N-tuple perpendicularly stratified components with 1 (mod p_ν_) or 1 (non-mod) in the *v*-th coordinate and 0 (mod p_ν_) or 0 (non-mod) in all other coordinates.

Here, a standard basis for M (= {Z_p_ or C ^×N^, ◊}) is denoted by a set of N-tuple basis vectors [e_(1),_ e_(2),_ e_(3),…,_ e_(ν),…,_e_(N)_] (= φ_N_) [9, 20–22]. Thus,

6 $$ {\mathrm{e}}_1=\kern0.5em \left[\begin{array}{l}1\kern1.5em \left( \mod \kern0.5em \mathrm{p}1\right)\\ {}0\kern1em \left( \mod \kern0.5em \mathrm{p}2\right)\\ {}0\\ {}\vdots \\ {}0\\ {}\vdots \\ {}0\end{array}\right],\kern0.5em {\mathrm{e}}_2=\kern0.5em \left[\begin{array}{l}0\kern1.5em \left( \mod \kern0.5em \mathrm{p}1\right)\\ {}1\kern1.5em \left( \mod \kern0.5em \mathrm{p}2\right)\\ {}0\\ {}\vdots \\ {}0\\ {}\vdots \\ {}0\end{array}\right],\kern0.5em {\mathrm{e}}_3=\left[\begin{array}{l}0\\ {}0\\ {}1\\ {}\vdots \\ {}0\\ {}\vdots \\ {}0\end{array}\right],\dots, \kern0.5em {\mathrm{e}}_{\mathrm{v}}=\left[\begin{array}{l}0\\ {}0\\ {}0\\ {}\vdots \\ {}1\;\left(\upnu -\mathrm{t}\mathrm{h}\;\mathrm{layer}\right)\\ {}\vdots \\ {}0\end{array}\right],\dots, \kern0.5em {\mathrm{e}}_{\mathrm{N}}\kern0.5em =\left[\begin{array}{l}0\\ {}0\\ {}0\\ {}\vdots \\ {}0\\ {}\vdots \\ {}1\end{array}\right]\;. $$

Expression (6) is a conventional vector expression of the operational rules (3) acting on the *v*-th vector e_(ν)_.

Now, let V be a vector space over the field C of complex numbers and GL(V) be the group of isomorphisms of V onto itself. By definition, an element *a* of GL(V) is a linear mapping of V into V via its inverse *a*^-1^. When V has a finite basis (e_(ν)_) of *n* elements, each linear map a: V → V is defined by a square matrix (a_st_) of order *n*. The images a(e_t_) in terms of the basis (e_s_) are given by

7 $$ \mathrm{a}\left({\mathrm{e}}_{\mathrm{t}}\right)=\sum_{\mathrm{s}}{\mathrm{a}}_{\mathrm{s}\mathrm{t}}{\mathrm{e}}_{\mathrm{s}}. $$

Thus, the group GL(V) is the group of invertible square matrices of order *n* [22]. Suppose that G is a finite group with identity element 1 and composition (j, k) ↦ jk. A linear representation of G in V is a homomorphism ρ from group G into group GL(V).

8 $$ \uprho :\ \mathrm{G}\to \mathrm{GL}\left(\mathrm{V}\right). $$

To each element j ∈ G, we associate an element ρ(j) of GL(V) such that

9 $$ \uprho \left(\mathrm{j}\mathrm{k}\right) = \uprho \left(\mathrm{j}\right)\cdotp \uprho \left(\mathrm{k}\right)\ \mathrm{f}\mathrm{o}\mathrm{r}\ \mathrm{j},\ \mathrm{k}\kern0.5em \in \mathrm{G}. $$

Additionally,

10 $$ \uprho (1) = 1,\ \uprho \left({\mathrm{j}}^{-1}\right) = \uprho {\left(\mathrm{j}\right)}^{-1}. $$

When ρ is given, we say that V is a representation of G [22].

Moreover, if V has finite dimensions *n*, then *n* denotes the degree of the representation. Let e_(ν)_ be a basis of V (an orthonormal vector in the *v*-th layer), and D(R_j_) be the matrix of ρ(R_j_) with respect to this basis. In our model, j ≡ R_j_, k ≡ R_k_, and *n* = 1. The correspondences

11 $$ {\mathrm{R}}_{\mathrm{j}}:\mathrm{M}\to \mathrm{M}\ \mathrm{and}\kern0.5em \uprho :\ {\mathrm{R}}_{\mathrm{j}}\to \mathrm{D}\left({\mathrm{R}}_{\mathrm{j}}\right) $$

also apply. Hence, for arbitrary elements R_j_, R_k_ of M, we have

12 $$ \left({\mathrm{R}}_{\mathrm{j}}{\mathrm{R}}_{\mathrm{k}}\right)\mathrm{M} = {\mathrm{R}}_{\mathrm{j}}\left({\mathrm{R}}_{\mathrm{k}}\mathrm{M}\right),\ \mathrm{and}\ \mathrm{consequently}\kern0.5em \mathrm{D}\left({\mathrm{R}}_{\mathrm{j}}{\mathrm{R}}_{\mathrm{k}}\right)=\mathrm{D}\left({\mathrm{R}}_{\mathrm{j}}\right)\mathrm{D}\left({\mathrm{R}}_{\mathrm{k}}\right). $$

Group M can then be expressed as the product of matrices R_j_ and R_k_.

Denoting by D_st_(R_j_) the coefficients of the matrix D(R_j_), (12) becomes [22]

13 $$ {\mathrm{D}}_{\mathrm{st}}\left({\mathrm{R}}_{\mathrm{j}}{\mathrm{R}}_{\mathrm{k}}\right)=\sum_{\mathrm{u}}{\mathrm{D}}_{\mathrm{su}}\left({\mathrm{R}}_{\mathrm{j}}\right)\cdot \mathrm{D}{}_{\mathrm{u}}{}_{\mathrm{t}}\left({\mathrm{R}}_{\mathrm{k}}\right). $$

In our model, the components are discriminated by the stratified form, which is incompatible with the interactive rules between elements of a matrix. The above analysis is performed on a square matrix of order *n* = 1 in Eqs. (4)-(13).

A vector space of definable multiplication (additive group) is called a K-algebra (ring). To confirm that the K-vector space is a K-algebra, we denote a linear transformation of V as V → V, and a set of entire endomorphisms as End_K_(V). For *n*-dimensional V, End_K_(V) can be viewed as an (*n* × *n*) K-element matrix Mat_K_(*n*). Then, for f, g ∈ End_K_(V), the product ‘f◦g’ is defined as ‘fg’, and the multiplication is defined for End_K_(V). Therefore, End_K_(V) is an *n*-dimensional K-algebra, and can be viewed as a matrix ring [22].

We next find the C[M] module that satisfies the following postulates:

1. M is an additive group,
2. Multiplication by R is definable in M; that is, for a, b ∈ R (where R is a set of real numbers) and R_j_ ∈ M, we have
  14 $$ \mathrm{a}\left({\mathrm{bR}}_{\mathrm{j}}\right) = \left(\mathrm{ab}\right){\mathrm{R}}_{\mathrm{j}}\in \mathrm{M},\ 1\cdotp {\mathrm{R}}_{\mathrm{j}} = {\mathrm{R}}_{\mathrm{j}}. $$
3. 15 $$ \left(\mathrm{a} + \mathrm{b}\right)\ {\mathrm{R}}_{\mathrm{j}} = {\mathrm{aR}}_{\mathrm{j}} + {\mathrm{bR}}_{\mathrm{j}},\ \mathrm{a}\left({\mathrm{R}}_{\mathrm{j}} + {\mathrm{R}}_{\mathrm{k}}\right) = {\mathrm{aR}}_{\mathrm{j}} + {\mathrm{bR}}_{\mathrm{k}}. $$

Under these postulates, a pair of arbitrary elements R_j_ and R_k_ must be homomorphic.

To maintain incompatibility with the above C[M] module, we reinterpret the ◊ operation as ‘+’ or ‘×’prior to matrix manipulation. That is, the rule of non-modular/modular addition is simply denoted by ‘+’, and a composite ‘R_j_R_k_’ is treated as a simple multiplication ‘R_j_ × R_k_’. This treatment avoids ambiguity when ◊ is undetermined in the matrix multiplication.

First, we denote the representation matrix of an operator R_j_ by D(R_j_), where R_j_ can re-denote a 1-dimensional square matrix [R_j_] composed of N-tuple perpendicularly stratified components of r_(j)ν_ (*v* = 1,2,…N) over basis φ_N_. Then, denoting R_j_e_(ν)_ as a basis e_(ν)_ multiplied by R_j_, the representation matrix D(R_j_) is expressed as follows:

16 $$ {\mathrm{R}}_{\mathrm{j}}{\upvarphi}_{\mathrm{N}} = \left[{\mathrm{R}}_{\mathrm{j}}{\mathrm{e}}_{(1)},\ {\mathrm{R}}_{\mathrm{j}}{\mathrm{e}}_{(2)},\dots, {\mathrm{R}}_{\mathrm{j}}{{\mathrm{e}}_{\Big(}}_{\upnu \Big)},\dots, {\mathrm{R}}_{\mathrm{j}}{\mathrm{e}}_{\left(\mathrm{N}\right)}\left] = \right[{\mathrm{e}}_{(1)},{\mathrm{e}}_{(2)},\dots, {{\mathrm{e}}_{\Big(}}_{\upnu \Big)},\dots, {\mathrm{e}}_{\left(\mathrm{N}\right)}\right]\mathrm{D}\left({\mathrm{R}}_{\mathrm{j}}\right) = {\upvarphi}_{\mathrm{N}}\mathrm{D}\left({\mathrm{R}}_{\mathrm{j}}\right). $$

By Eqs. (4) and (6), the left–hand side of Eq. (16) becomes

17 $$ \left[{\mathrm{R}}_{\mathrm{j}}{\mathrm{e}}_{(1)},{\mathrm{R}}_{\mathrm{j}}{\mathrm{e}}_{(2)},\dots, {\mathrm{R}}_{\mathrm{j}}{{\mathrm{e}}_{(}}_{\upnu )},\dots, {\mathrm{R}}_{\mathrm{j}}{\mathrm{e}}_{\left(\mathrm{N}\right)}\right]=\left[{\mathrm{e}}_{(1)},{\mathrm{e}}_{(2)},\dots, {{\mathrm{e}}_{(}}_{\upnu )},\dots, {\mathrm{e}}_{\left(\mathrm{N}\right)}\right]\left[{\mathrm{R}}_{\mathrm{j}}\right]. $$

Hence,

18 $$ \mathrm{D}\left({\mathrm{R}}_{\mathrm{j}}\right) = \left[{\mathrm{R}}_{\mathrm{j}}\right]. $$

The one-dimensional square matrix [R_j_] is equivalent to the perpendicularly stratified vector R_j_. In essence, (17) means the combination

19 $$ \left[{\mathrm{R}}_{\mathrm{j}}{\mathrm{e}}_{\left(\upnu \right)}\right] = \left[{\mathrm{e}}_{\left(\upnu \right)}\right]\left[{\mathrm{R}}_{\mathrm{j}}\right] $$

in the *v*-th layer, where *ν* = 1, 2,…, N. Referring to Eq. (6), we observe that [R_j_e_(ν)_] = [1][R_j_] in the *v*-th layer, and [R_j_e_(ν)_] = [0][R_j_] in the *μ*-th layer (*μ* ≠ *ν*). In subsequent sections, we construct the perpendicularly stratified vectors into multi-dimensional square matrices.

#### §2. Further application of stratified vectors as elements of a larger square matrix

Here, we incorporate the stratified vectors R_j_ in set M into a larger matrix. As discussed in our previous paper, we consider that our approach will assist the treatment of a patient’s disease state R_j_ [19]. We also suggest solutions in the absence of a practical group matrix representation.

The order |M| of a set M might be infinite. If |M| = ∞, we can apply an operation τ that changes the *m*-tuple combination of arbitrary R_j_s from M to the same number of combination of R_j_s (where *m* is a positive integer not exceeding |M|; *m* = 1, 2, 3,…|M|). Let τ be a mapping such that

20 $$ \uptau :\left[\begin{array}{c}\hfill \mathrm{R}\mathrm{a}\hfill \\ {}\hfill \mathrm{R}\mathrm{b}\hfill \\ {}\hfill \mathrm{R}\mathrm{c}\hfill \\ {}\hfill \vdots \hfill \\ {}\hfill \mathrm{R}\mathrm{d}\hfill \end{array}\right]\left(\equiv {\mathrm{R}}^{\mathrm{m}}\right)\to \left[\begin{array}{c}\hfill {\mathrm{R}\mathrm{a}}^{\hbox{'}}\hfill \\ {}\hfill {\mathrm{R}\mathrm{b}}^{\hbox{'}}\hfill \\ {}\hfill {\mathrm{R}\mathrm{c}}^{\hbox{'}}\hfill \\ {}\hfill \vdots \hfill \\ {}\hfill {\mathrm{R}\mathrm{d}}^{\hbox{'}}\hfill \end{array}\right]\left(\equiv {{\mathrm{R}}^{\mathrm{m}}}^{\hbox{'}}\right)\kern0.5em \left(m-\mathrm{row},\ 1-\mathrm{column},\ \mathrm{N}\hbox{-} \mathrm{layer}\ \mathrm{matrix}\right). $$

Note that τ is a mapping from a *m*-tuple R_j_ to another *m*-tuple R_j_, where the R_j_s are freely selected from M, and infinite τ’s are permitted, because at this stage we merely introduce our perpendicular approach. In practice, τ could be an intervention/treatment T administered throughout the clinical course of a certain patient.

As a more tangible example, suppose that Eq. (1) represents a series of disease states of a certain patient over the entire treatment course; namely, R_1_(=R_(0→1)_)◊R_(1→2)_◊R_(2→3)_◊…◊R_(m-2→m-1)_◊R_(m-1→m)_ = R_m_ (where R_0_ is the initial state of the patient; for consistency of expression, we use R_(0→1)_ rather than R_1_ until now). This sequence could also be written as a series of row vectors

21 $$ \left[{\mathrm{R}}_{\left(0\to 1\right)}\lozenge {{\mathrm{R}}_{\Big(1}}_{\to 2\Big)}\lozenge {{\mathrm{R}}_{\Big(2}}_{\to 3\Big)}\lozenge \dots \lozenge {{\mathrm{R}}_{\Big(\mathrm{m}-2}}_{\to \mathrm{m}-1\Big)}\lozenge {{\mathrm{R}}_{\Big(\mathrm{m}-1}}_{\to \mathrm{m}\Big)}\right], $$

where each R_j_ is a component of a row vector, and the ◊s are optionally included for their explicit recognition. That is, we can regard (21) as (*m* × 1) N-tuple perpendicular vectors

22 $$ \left[{{\mathrm{R}}_{\Big(0}}_{\to 1\Big)},{{\mathrm{R}}_{\Big(1}}_{\to 2\Big)},{{\mathrm{R}}_{\Big(2}}_{\to 3\Big)},\dots, {{\mathrm{R}}_{\Big(\mathrm{m}-2}}_{\to \mathrm{m}-1\Big)},{{\mathrm{R}}_{\Big(\mathrm{m}-1}}_{\to \mathrm{m}\Big)}\right]. $$

Likewise, (20) can be assembled from column vectors obtained by transposing (21), as displayed in (23). In this regard, provided that τ is a mapping for a treatment T of the patient, and R^m^ is the entire course of the untreated patient, the R^m'^ (20) of the patient undergoing treatment T becomes

23 $$ \uptau :\kern0.5em \left[\begin{array}{c}\hfill \mathrm{R}\left(0\to 1\right)\hfill \\ {}\hfill \lozenge \hfill \\ {}\hfill \mathrm{R}\left(1\to 2\right)\hfill \\ {}\hfill \lozenge \hfill \\ {}\hfill \mathrm{R}\left(2\to 3\right)\hfill \\ {}\hfill \lozenge \hfill \\ {}\hfill \vdots \hfill \\ {}\hfill \lozenge \hfill \\ {}\hfill \mathrm{R}\left(\mathrm{m}-2\to \mathrm{m}-1\right)\hfill \\ {}\hfill \lozenge \hfill \\ {}\hfill \mathrm{R}\left(\mathrm{m}-1\to \mathrm{m}\right)\hfill \end{array}\right]\left(\equiv {\mathrm{R}}^{\mathrm{m}}\right)\to \left[\begin{array}{c}\hfill \mathrm{R}{\left(0\to 1\right)}^{\hbox{'}}\hfill \\ {}\hfill \lozenge \hfill \\ {}\hfill \mathrm{R}{\left(1\to 2\right)}^{\hbox{'}}\hfill \\ {}\hfill \lozenge \hfill \\ {}\hfill \mathrm{R}{\left(2\to 3\right)}^{\hbox{'}}\hfill \\ {}\hfill \lozenge \hfill \\ {}\hfill \vdots \hfill \\ {}\hfill \lozenge \hfill \\ {}\hfill \mathrm{R}{\left(\mathrm{m}-2\to \mathrm{m}-1\right)}^{\hbox{'}}\hfill \\ {}\hfill \lozenge \hfill \\ {}\hfill \mathrm{R}{\left(\mathrm{m}-1\to \mathrm{m}\right)}^{\hbox{'}}\hfill \end{array}\right]\left(\equiv {{\mathrm{R}}^{\mathrm{m}}}^{\hbox{'}}\right)\kern0.5em \left(m-\mathrm{row},\ 1\hbox{-} \mathrm{column},\ \mathrm{N}\hbox{-} \mathrm{layer}\ \mathrm{space}\ \mathrm{matrix}\right). $$

Both of R^m^ and R^m'^ can be confirmed as *m*-tuple products of M (≡ M^×m^). Additionally, R^m^ and R^m'^ are N-tuple perpendicularly stratified vectors, implying that (18) can be similarly expressed in stratified form.

From (20), there exists a mapping π (∈ GL(V^m^), an *m*-dimensional linear space):

24 $$ {\mathrm{R}}^{\mathrm{m}}\to {{\mathrm{R}}^{\mathrm{m}}}^{\hbox{'}}. $$

Let g (∈ GL(V^m^)) satisfy

25 $$ {\mathrm{gR}}^{\mathrm{m}}\kern0.5em =\kern0.5em {{\mathrm{R}}^{\mathrm{m}}}^{\hbox{'}}. $$

g (treatment T on the patient) is an *m*-dimensional, N-tuple perpendicularly stratified square matrix $\left[{}_{\mathrm{m}}^{\mathrm{N}}\mathrm{D}\left(\mathrm{g}\right)\right]$ satisfying the relationship:

26 $$ {{\mathrm{R}}^{\mathrm{m}}}^{\hbox{'}}=\left[{}_{\mathrm{m}}^{\mathrm{N}}\mathrm{D}\left(\mathrm{g}\right)\right]{\mathrm{R}}^{\mathrm{m}}, $$

which is expressed as ordinal matrix products. The *v*-th layer of the matrix $\left[{}_{\mathrm{m}}^{\mathrm{N}}\mathrm{D}\left(\mathrm{g}\right)\right]$ is denoted by ${\left[{}_{\mathrm{m}}^{\mathrm{N}}\mathrm{D}\left(\mathrm{g}\right)\right]}_{\upnu}$, by which (26) becomes

27 $$ {\mathrm{R}}_{\upnu}^{\mathrm{m}\hbox{'}}={\left[{}_{\mathrm{m}}^{\mathrm{N}}\mathrm{D}\left(\mathrm{g}\right)\right]}_{\upnu}\kern0ex {\mathrm{R}}_{\upnu}^{\mathrm{m}}, $$

or ‹ R^m'^ › _ν_ = ‹ $$ \left[{}_{\mathrm{m}}^{\mathrm{N}}\mathrm{D}\left(\mathrm{g}\right)\right] $$ › _ν_ ‹ R^m^ › _ν_. Equivalently,

28 $$ {\left[\begin{array}{c}\hfill \mathrm{R}{\left(0\to 1\right)}^{\hbox{'}}\hfill \\ {}\hfill \lozenge \hfill \\ {}\hfill \mathrm{R}{\left(1\to 2\right)}^{\hbox{'}}\hfill \\ {}\hfill \lozenge \hfill \\ {}\hfill \mathrm{R}{\left(2\to 3\right)}^{\hbox{'}}\hfill \\ {}\hfill \lozenge \hfill \\ {}\hfill \vdots \hfill \\ {}\hfill \lozenge \hfill \\ {}\hfill \mathrm{R}{\left(\mathrm{m}-2\to \mathrm{m}-1\right)}^{\hbox{'}}\hfill \\ {}\hfill \lozenge \hfill \\ {}\hfill \mathrm{R}{\left(\mathrm{m}-1\to \mathrm{m}\right)}^{\hbox{'}}\hfill \end{array}\right]}_{\left(\upnu \right)}={\left[{}_{\mathrm{m}}{}^{\mathrm{N}}\mathrm{D}\left(\mathrm{g}\right)\right]}_{\left(\upnu \right)}\kern0.5em {\left[\begin{array}{c}\hfill \mathrm{R}\left(0\to 1\right)\hfill \\ {}\hfill \lozenge \hfill \\ {}\hfill \mathrm{R}\left(1\to 2\right)\hfill \\ {}\hfill \lozenge \hfill \\ {}\hfill \mathrm{R}\left(2\to 3\right)\hfill \\ {}\hfill \lozenge \hfill \\ {}\hfill \vdots \hfill \\ {}\hfill \lozenge \hfill \\ {}\hfill \mathrm{R}\left(\mathrm{m}-2\to \mathrm{m}-1\right)\hfill \\ {}\hfill \lozenge \hfill \\ {}\hfill \mathrm{R}\left(\mathrm{m}-1\to \mathrm{m}\right)\hfill \end{array}\right]}_{\left(\upnu \right)} $$

29 $$ =\left[\begin{array}{ccccc}\hfill {\mathrm{D}}_{\left(\upnu \right)11}\hfill & \hfill {\mathrm{D}}_{\left(\upnu \right)12}\hfill & \hfill {\mathrm{D}}_{\left(\upnu \right)13}\hfill & \hfill \cdots \hfill & \hfill {\mathrm{D}}_{\left(\upnu \right)1\mathrm{m}}\hfill \\ {}\hfill {\mathrm{D}}_{\left(\upnu \right)21}\hfill & \hfill {\mathrm{D}}_{\left(\upnu \right)22}\hfill & \hfill {\mathrm{D}}_{\left(\upnu \right)23}\hfill & \hfill \cdots \hfill & \hfill {\mathrm{D}}_{\left(\upnu \right)2\mathrm{m}}\hfill \\ {}\hfill {\mathrm{D}}_{\left(\upnu \right)31}\hfill & \hfill {\mathrm{D}}_{\left(\upnu \right)32}\hfill & \hfill {\mathrm{D}}_{\left(\upnu \right)33}\hfill & \hfill \cdots \hfill & \hfill {\mathrm{D}}_{\left(\upnu \right)3\mathrm{m}}\hfill \\ {}\hfill \vdots \hfill & \hfill \vdots \hfill & \hfill \vdots \hfill & \hfill \ddots \hfill & \hfill \vdots \hfill \\ {}\hfill {\mathrm{D}}_{\left(\upnu \right)\mathrm{m}1}\hfill & \hfill {\mathrm{D}}_{\left(\upnu \right)\mathrm{m}2}\hfill & \hfill {\mathrm{D}}_{\left(\upnu \right)\mathrm{m}3}\hfill & \hfill \cdots \hfill & \hfill {\mathrm{D}}_{\left(\upnu \right)\mathrm{m}\mathrm{m}}\hfill \end{array}\cdots \right]\left[\begin{array}{c}\hfill \mathrm{R}\left(0\to 1\right)\hfill \\ {}\hfill \lozenge \hfill \\ {}\hfill \mathrm{R}\left(1\to 2\right)\hfill \\ {}\hfill \lozenge \hfill \\ {}\hfill \mathrm{R}\left(2\to 3\right)\hfill \\ {}\hfill \lozenge \hfill \\ {}\hfill \vdots \hfill \\ {}\hfill \lozenge \hfill \\ {}\hfill \mathrm{R}\left(\mathrm{m}-2\to \mathrm{m}-1\right)\hfill \\ {}\hfill \lozenge \hfill \\ {}\hfill \mathrm{R}\left(\mathrm{m}-1\to \mathrm{m}\right)\hfill \end{array}\right]\left(\upnu \right) $$

The elements in (29) are the individual components in the cross-section of the *ν*-th layer; ‹R_a_›_ν_ = r_(a)ν_. Like the elements R_j_, D_(ν)st_ denotes an element (∈ M) of $\left[{}_{\mathrm{m}}^{\mathrm{N}}\mathrm{D}\left(\mathrm{g}\right)\right]$ in the *s*-th row and *t*-th column of the *v*-th layer (*v* = 1,2,…,N). Note that (23), (28) and (29) express not only ordinal matrix relationships; that is, linear combinations of respective components in R^m^ and R^m'^, but also the clinical course (1) of the patient through a series of ◊ operations. However, including the ◊s in the row/column vector is optional, and the row/column vectors (21) and (22) are strictly equivalent. Naturally, (28) and (29) can be transposed through [_m_^N^D(g)]^t^, the transposed matrix of $\left[{}_{\mathrm{m}}^{\mathrm{N}}\mathrm{D}\left(\mathrm{g}\right)\right]$.

30 $$ \begin{array}{l}\left[\begin{array}{ccccccccccc}\hfill \mathrm{R}{\left(0\to 1\right)}^{\hbox{'}}\hfill & \hfill \lozenge \hfill & \hfill \mathrm{R}{\left(1\to 2\right)}^{\hbox{'}}\hfill & \hfill \lozenge \hfill & \hfill \mathrm{R}{\left(2\to 3\right)}^{\hbox{'}}\hfill & \hfill \lozenge \hfill & \hfill \dots \hfill & \hfill \lozenge \hfill & \hfill \mathrm{R}{\left(\mathrm{m}-2\to \mathrm{m}-1\right)}^{\hbox{'}}\hfill & \hfill \lozenge \hfill & \hfill \mathrm{R}{\left(\mathrm{m}-1\to \mathrm{m}\right)}^{\hbox{'}}\hfill \end{array}\right]\\ {}=\left[{\mathrm{R}}_{\left(0\to 1\right)}\lozenge {{\mathrm{R}}_{\Big(1}}_{\to 2\Big)}\lozenge {{\mathrm{R}}_{\Big(2}}_{\to 3\Big)}\lozenge \dots \lozenge {{\mathrm{R}}_{\Big(\mathrm{m}-2}}_{\to \mathrm{m}-1\Big)}\lozenge {{\mathrm{R}}_{\Big(\mathrm{m}-1}}_{\to \mathrm{m}\Big)}\right]{\left[{}_{\mathrm{m}}{}^{\mathrm{N}}\mathrm{D}\left(\mathrm{g}\right)\right]}^{\mathrm{t}}.\end{array} $$

Expressions (28)–(30) are optional vector descriptions representing the change in the pre/post-state of a patient undergoing an intervention/treatment T, which preserves the operational meaning. The notation now takes a double meaning, representing 1) a series of changes from the previous state R_(j-1)_ to a subsequent state R_j_ (*j* = 1, 2,…, *m*), where the change at session *j* occurs by an operation R_(j-1→j)_ to R_(j-1)_, or 2) the post-state of the intervention/treatment T, resulting from the linear combination of the pre-state and the subsequently modified states R_(j-1→j)_. We envisage that this notation could concisely represent the multiple aspects of a patient’s clinical history.

In (28)–(30), the operator ◊ plays a negligible role, and we can omit the ◊s from an arbitrarily ordered column vector as follows:

31 $$ {\mathrm{R}}^{\mathrm{m}} = \kern0.5em \left[\begin{array}{c}\hfill \mathrm{R}\left(0\to 1\right)\hfill \\ {}\hfill \lozenge \hfill \\ {}\hfill \mathrm{R}\left(3\to 4\right)\hfill \\ {}\hfill \lozenge \hfill \\ {}\hfill \mathrm{R}\left(7\to 2\right)\hfill \\ {}\hfill \lozenge \hfill \\ {}\hfill \vdots \hfill \\ {}\hfill \lozenge \hfill \\ {}\hfill \mathrm{R}\left(\mathrm{a}\to \mathrm{b}\right)\hfill \\ {}\hfill \lozenge \hfill \\ {}\hfill \mathrm{R}\left(\mathrm{c}\to \mathrm{d}\right)\hfill \end{array}\right] = \kern0.5em \left[\begin{array}{c}\hfill \mathrm{R}\left(0\to 1\right)\hfill \\ {}\hfill \hfill \\ {}\hfill \mathrm{R}\left(3\to 4\right)\hfill \\ {}\hfill \hfill \\ {}\hfill \mathrm{R}\left(7\to 2\right)\hfill \\ {}\hfill \hfill \\ {}\hfill \vdots \hfill \\ {}\hfill \hfill \\ {}\hfill \mathrm{R}\left(\mathrm{a}\to \mathrm{b}\right)\hfill \\ {}\hfill \hfill \\ {}\hfill \mathrm{R}\left(\mathrm{c}\to \mathrm{d}\right)\hfill \end{array}\right]. $$

This alternative representation of (28) and (29) is naturally permissible, and both stratified vectors in (31) can be equivalently treated in ordinal vector and matrix operations.

All of the elements in (29) are N-tuple perpendicularly stratified vectors (∈ set M), thus confirming the matrix representation of $$ \left[{}_{\mathrm{m}}^{\mathrm{N}}\mathrm{D}\left(\mathrm{g}\right)\right] $$$$ (\equiv {\mathrm{M}}^{{\times \mathrm{m}}^2}; $$ m^2^-tuple product of M).

Similar to (24)–(29), a series of changes such as ${\mathrm{R}}^{\mathrm{m}}\kern0.5em \overset{\mathrm{g}1}{\to}\kern0.5em {{\mathrm{R}}^{\mathrm{m}}}^{\hbox{'}}\kern0.5em \overset{\mathrm{g}2}{\to}\kern0.5em {{\mathrm{R}}^{\mathrm{m}}}^{"}\kern0.5em \overset{\mathrm{g}3}{\to}\dots \overset{\mathrm{g}\left(\mathrm{n}-1\right)}{\to}\kern0.5em {{\mathrm{R}}^{\mathrm{m}}}^{\left(\mathrm{n}\right)},$ where g_1_, g_2_,…,g_(n-2)_, g_(n-1)_ (∈ GL(V^m^)) satisfy:

32 $$ {\mathrm{g}}_{\mathrm{i}}{\mathrm{R}}^{\mathrm{m}}={{\mathrm{R}}^{\mathrm{m}}}^{\hbox{'}},\kern0.5em \mathrm{and}\kern0.5em {{\mathrm{R}}^{\mathrm{m}}}^{\hbox{'}}=\left[{}_{\mathrm{m}}^{\mathrm{N}}\mathrm{D}\left({\mathrm{g}}_{\mathrm{i}}\right)\right]{\mathrm{R}}^{\mathrm{m}}\kern0.5em \left(i=1,2,\dots, n-1\right) $$

can be written as combinations of

33 $$ {{\mathrm{R}}^{\mathrm{m}}}^{{}^{\left(\mathrm{n}\right)}}=\left[{}_{\mathrm{m}}^{\mathrm{N}}\mathrm{D}\left({\mathrm{g}}_{\left(\mathrm{n}-1\right)}\right)\right]\dots \left[{}_{\mathrm{m}}^{\mathrm{N}}\mathrm{D}\left({\mathrm{g}}_3\right)\right]\left[{}_{\mathrm{m}}^{\mathrm{N}}\mathrm{D}\left({\mathrm{g}}_2\right)\right]\left[{}_{\mathrm{m}}^{\mathrm{N}}\mathrm{D}\left({\mathrm{g}}_1\right)\right]{\mathrm{R}}^{\mathrm{m}} $$

and

34 $$ {\mathrm{R}}_{\upnu}^{{\mathrm{m}}^{\left(\mathrm{n}\right)}}={\left[{}_{\mathrm{m}}^{\mathrm{N}}\mathrm{D}\left({\mathrm{g}}_{\left(\mathrm{n}-1\right)}\right)\right]}_{\upnu}\dots {\left[{}_{\mathrm{m}}^{\mathrm{N}}\mathrm{D}\left({\mathrm{g}}_3\right)\right]}_{\upnu}{\left[{}_{\mathrm{m}}^{\mathrm{N}}\mathrm{D}\left({\mathrm{g}}_2\right)\right]}_{\upnu}{\left[{}_{\mathrm{m}}^{\mathrm{N}}\mathrm{D}\left({\mathrm{g}}_1\right)\right]}_{\upnu}{\mathrm{R}}_{\upnu}^{\mathrm{m}}. $$

The post-interventional state ${\mathrm{R}}_0^{\mathrm{m}\hbox{'}}$ is related to the pre-interventional state ${\mathrm{R}}_0^{\mathrm{m}}$ as follows:

R_0_^m '^ = [_m_^N^D(g_(n − 1)_)]_0_ … [_m_^N^D(g_3_)]_0_[_m_^N^D(g_2_)]_0_[_m_^N^D(g_1_)]_0_R_0_^m^, where ‘$\left[{}_{\mathrm{m}}^{\mathrm{N}}\mathrm{D}\left(\mathrm{g}\right)\right]$_0_ = _m_I_(0)_ (*m* × *m* identity matrix)’. We emphasize that ${\mathrm{R}}_0^{\mathrm{m}}$ should be discriminated from the identity state R_0_ (containing all zeros) described in our previous articles [18, 19].

To compose a basis of orthonormal vectors, we express the 1 × *m* unit vectors as in (6), but these should be viewed as column vectors in the plane of this paper, which represent the *v*-th cross-section. Because the operational units are defined for each layer, all of the components obey the same operational rules, different from the case illustrated in (6).

The basis is denoted by a set of m-tuple column vectors in the *v*-th layer

35 $$ \left[{\mathrm{e}}_{\left(\upnu \right)1},{\mathrm{e}}_{\left(\upnu \right)2},{\mathrm{e}}_{\left(\upnu \right)3},\dots, {\mathrm{e}}_{\left(\upnu \right)\upmu},\dots, {\mathrm{e}}_{\left(\upnu \right)\mathrm{m}}\right]\left(={\varphi}_{\left(\upnu \right)\mathrm{m}}\right), $$

where e_(ν)μ_ (*m*-row, 1-column, N-layer) (*μ* = 1, 2,…, *m*) is a basis vector with 1 in the *μ*-th coordinate of the *v*-th layer, and 0 otherwise. Similarly, e_(ν)μ_ (*μ* = 1, 2,…, *m*) contains 0 in all but the *μ*-th coordinate of the *v*-th layer. (##)

36 $$ {\mathrm{e}}_{\left(\upnu \right)1}={\left[\begin{array}{l}1\\ {}0\\ {}0\\ {}\vdots \\ {}0\\ {}\vdots \\ {}0\end{array}\right]}_{\left(\upnu \right)},\ {\mathrm{e}}_{\left(\upnu \right)2}={\left[\begin{array}{l}0\\ {}1\\ {}0\\ {}\vdots \\ {}0\\ {}\vdots \\ {}0\end{array}\right]}_{\left(\upnu \right)},\ {\mathrm{e}}_{\left(\upnu \right)3}={\left[\begin{array}{l}0\\ {}0\\ {}1\\ {}\vdots \\ {}0\\ {}\vdots \\ {}0\end{array}\right]}_{\left(\upnu \right)},\dots,\ {\mathrm{e}}_{\left(\upnu \right)\upmu}={\left[\begin{array}{l}0\\ {}0\\ {}0\\ {}\vdots \\ {}1\;\left(\upmu -\mathrm{t}\mathrm{h}\;\mathrm{row}\right)\\ {}\vdots \\ {}0\end{array}\right]}_{\left(\upnu \right)},\dots,\ {\mathrm{e}}_{\left(\upnu \right)\mathrm{m}}={\left[\begin{array}{l}0\\ {}0\\ {}0\\ {}\vdots \\ {}0\\ {}\vdots \\ {}1\end{array}\right]}_{\left(\upnu \right)} $$

(cross-section at the *v*-th layer).

(36) comprises the following *m* × *m* identity matrix:

37 $$ {}_{\mathrm{m}}{\mathrm{I}}_{\left(\upnu \right)}={\left[\begin{array}{cccc}\hfill 1\hfill & \hfill 0\hfill & \hfill \cdots \hfill & \hfill 0\hfill \\ {}\hfill 0\hfill & \hfill 1\hfill & \hfill \cdots \hfill & \hfill 0\hfill \\ {}\hfill \vdots \hfill & \hfill \vdots \hfill & \hfill \ddots \hfill & \hfill \vdots \hfill \\ {}\hfill 0\hfill & \hfill 0\hfill & \hfill \cdots \hfill & \hfill 1\hfill \end{array}\right]}_{\left(\upnu \right)}\kern0.5em \left(\mathrm{n}\mathrm{o}\mathrm{n}\hbox{-} \mathrm{modular}\ \mathrm{case}\right)\ \left(v = 1,\ 2,\dots,\ \mathrm{N}\right). $$

In this case, the cross-section at the *v*-th layer is non-modular.

38 $$ {}_{\mathrm{m}}{\mathrm{I}}_{\left(\upnu \right)}={\left[\begin{array}{cccc}\hfill 1\hfill & \hfill 0\hfill & \hfill \cdots \hfill & \hfill 0\hfill \\ {}\hfill 0\hfill & \hfill 1\hfill & \hfill \cdots \hfill & \hfill 0\hfill \\ {}\hfill \vdots \hfill & \hfill \vdots \hfill & \hfill \ddots \hfill & \hfill \vdots \hfill \\ {}\hfill 0\hfill & \hfill 0\hfill & \hfill \cdots \hfill & \hfill 1\hfill \end{array}\right]}_{\left(\upnu \right)}\kern0.5em \left( \mod \kern0.5em {\mathrm{p}}_{\upnu}\right)\ \left(v = 1,\ 2,\dots,\ \mathrm{N}\right). $$

In (38), the cross-section at the *v*-th layer is mod p_*v*_. In both (37) and (38), the operational unit is uniquely applied in the *v*-th layer. Then, (35) and (36) are equivalent and the compositions of (37) and (38) follow from the definition of operation in the *v*-th layer.

The matrices (27)–(29) can also be expressed in terms of the basis φ_(ν)m_. For this purpose, we define the image of element g (25) on φ_(ν)m_ as gφ_(ν)m_, and that on e_(ν)μ_ as ge_(ν)μ_; that is

39 $$ \begin{array}{l}\mathrm{g}{\upvarphi}_{\left(\upnu \right)\mathrm{m}} = \left[{\mathrm{ge}}_{\left(\upnu \right)1},{\mathrm{ge}}_{\left(\upnu \right)2},\dots, {\mathrm{ge}}_{\left(\upnu \right)\upmu},\dots, {\mathrm{ge}}_{\left(\upnu \right)\mathrm{m}}\left] = \right[{\mathrm{e}}_{\left(\upnu \right)1},{\mathrm{e}}_{\left(\upnu \right)2},\dots, {\mathrm{e}}_{\left(\upnu \right)\upmu},\dots, {\mathrm{e}}_{\left(\upnu \right)\mathrm{m}}\right]{\left[{}_{\mathrm{m}}{}^{\mathrm{N}}\mathrm{D}\left(\mathrm{g}\right)\right]}_{\upnu},\\ {}\left(\mathrm{where}\ v = 1,\ 2,\dots,\ \mathrm{N}\right).\end{array} $$

Note that each φ_(ν)m_ independently acts on an individual layer *v* (where *v* = 1, 2,…, N), and there exists a N-tuple product of φ_(ν)m_ (*v* = 1, 2,…, N). Therefore, we can express Φ_(N)_ as follows:

40 $$ \begin{array}{l}{\Phi}_{\left(\mathrm{N}\right)} = \left[{\upvarphi}_{(1)\mathrm{m}},{\upvarphi}_{(2)\mathrm{m}},\dots, {\upvarphi}_{\left(\upnu \right)\mathrm{m}},\dots, {\upvarphi}_{\left(\mathrm{N}\right)\mathrm{m}}\right]\ \left(v\kern0.5em  = 1,\ 2,\dots,\ \mathrm{N}\right)\\ {}=\left[\left[{\mathrm{e}}_{(1)1},{\mathrm{e}}_{(1)2}{{,\dots, \mathrm{e}}_{(1)}}_{\upmu},\dots, {\mathrm{e}}_{(1)\mathrm{m}}\left],\right[{\mathrm{e}}_{(2)1},{\mathrm{e}}_{(2)2}{{,\dots, \mathrm{e}}_{(2)}}_{\upmu},\dots, {\mathrm{e}}_{(2)\mathrm{m}}\left],\dots, \right[{{\mathrm{e}}_{\Big(}}_{\upnu \Big)1}{{,\mathrm{e}}_{\Big(}}_{\upnu \Big)2}{{,\dots, \mathrm{e}}_{\Big(}}_{\upnu \Big)\upmu}{{,\dots, \mathrm{e}}_{\Big(}}_{\upnu \Big)\mathrm{m}}\left],\dots, \right[{\mathrm{e}}_{\left(\mathrm{N}\right)1},{\mathrm{e}}_{\left(\mathrm{N}\right)2}{{,\dots, \mathrm{e}}_{\left(\mathrm{N}\right)}}_{\upmu},\dots, {\mathrm{e}}_{\left(\mathrm{N}\right)\mathrm{m}}\right]\right].\left(\mu = 1,2,\dots, m\right)\end{array} $$

Defining an image of element g on Φ_(N)_ as gΦ_(N)_, (39) is more concisely expressed as

41 $$ \begin{array}{l}{\mathrm{g}\Phi}_{\left(\mathrm{N}\right)} = \left[{\mathrm{g}\upvarphi}_{(1)\mathrm{m}},{\mathrm{g}\upvarphi}_{(2)\mathrm{m}},\dots, {\mathrm{g}\upvarphi}_{\left(\upnu \right)\mathrm{m}},\dots, {\mathrm{g}\upvarphi}_{\left(\mathrm{N}\right)\mathrm{m}}\right]\ \left(v = 1,\ 2,\dots,\ \mathrm{N}\right)\\ {} = \left[{\upvarphi}_{(1)\mathrm{m}},\kern0.5em {\upvarphi}_{(2)\mathrm{m}},\dots, \kern0.5em {\upvarphi}_{\left(\upnu \right)\mathrm{m}},\dots, {\upvarphi}_{\left(\mathrm{N}\right)\mathrm{m}}\right]\left[{}_{\mathrm{m}}{}^{\mathrm{N}}\mathrm{D},\left(\mathrm{g}\right)\right].\end{array} $$

The expanded form of (41) is

42 $$ =\left[\left[{\mathrm{ge}}_{(1)1},{\mathrm{ge}}_{(1)2},\dots, {\mathrm{ge}}_{(1)\upmu},\dots, {\mathrm{ge}}_{(1)\mathrm{m}}\right]\right[,{\mathrm{ge}}_{(2)1},{\mathrm{ge}}_{(2)2},\dots, {\mathrm{ge}}_{(2)\upmu},\dots, {\mathrm{ge}}_{(2)\mathrm{m}}\left],\dots, \right[{\mathrm{ge}}_{\left(\upnu \right)1},{\mathrm{ge}}_{\left(\upnu \right)2},\dots, {\mathrm{ge}}_{\left(\upnu \right)\upmu},\dots, {\mathrm{ge}}_{\left(\upnu \right)\mathrm{m}}\left],\dots, \right[{\mathrm{ge}}_{\left(\mathrm{N}\right)1},{\mathrm{ge}}_{\left(\mathrm{N}\right)2},\dots, {\mathrm{ge}}_{\left(\mathrm{N}\right)\upmu},\dots, {\mathrm{ge}}_{\left(\mathrm{N}\right)\mathrm{m}}\left]\right]\left[{}_{\mathrm{m}}{}^{\mathrm{N}}\mathrm{D},\left(\mathrm{g}\right)\right].\ \left(\mu = 1,\ 2,\dots,\ \mathrm{m}\right) $$

Moreover, for g_(n-1)_…g_3_g_2_g_1_ (∈ GL(V^m^), we have

43 $$ \begin{array}{l}\left({\mathrm{g}}_{\left(\mathrm{n}\hbox{-} 1\right)}\dots {\mathrm{g}}_3{\mathrm{g}}_2{\mathrm{g}}_1\right){\upvarphi}_{\left(\upnu \right)\mathrm{m}} = \left[\left({\mathrm{g}}_{\left(\mathrm{n}\hbox{-} 1\right)}\dots {\mathrm{g}}_3{\mathrm{g}}_2{\mathrm{g}}_1\right){\mathrm{e}}_{\left(\upnu \right)1},\left({\mathrm{g}}_{\left(\mathrm{n}\hbox{-} 1\right)}\dots {\mathrm{g}}_3{\mathrm{g}}_2{\mathrm{g}}_1\right){\mathrm{e}}_{\left(\upnu \right)2},\dots, \left({\mathrm{g}}_{\left(\mathrm{n}\hbox{-} 1\right)}\dots {\mathrm{g}}_3{\mathrm{g}}_2{\mathrm{g}}_1\right){\mathrm{e}}_{\left(\upnu \right)\upmu},\dots, \left({\mathrm{g}}_{\left(\mathrm{n}\hbox{-} 1\right)}\dots {\mathrm{g}}_3{\mathrm{g}}_2{\mathrm{g}}_1\right){\mathrm{e}}_{\left(\upnu \right)\mathrm{m}}\right]\\ {}=\left[{\mathrm{e}}_{\left(\upnu \right)1},{\mathrm{e}}_{\left(\upnu \right)2},\dots, {\mathrm{e}}_{\left(\upnu \right)\upmu},\dots, {\mathrm{e}}_{\left(\upnu \right)\mathrm{m}}\right]{\left[{}_{\mathrm{m}}{}^{\mathrm{N}}\mathrm{D}\left({\mathrm{g}}_{\left(\mathrm{n}\hbox{-} 1\right)}\right)\right]}_{\upnu}\dots {\left[{}_{\mathrm{m}}{}^{\mathrm{N}}\mathrm{D}\left({\mathrm{g}}_3\right)\right]}_{\upnu}{\left[{}_{\mathrm{m}}{}^{\mathrm{N}}\mathrm{D}\left({\mathrm{g}}_2\right)\right]}_{\upnu}{\left[{}_{\mathrm{m}}{}^{\mathrm{N}}\mathrm{D}\left({\mathrm{g}}_1\right)\right]}_{\upnu}.\ \left(n = 1,\ 2,\dots \right)\end{array} $$

Similarly, (43) can be integrated over all layers (*v* = 1, 2,…, N) as follows:

44 $$ \begin{array}{l}\left({\mathrm{g}}_{\left(\mathrm{n}\hbox{-} 1\right)}\dots {\mathrm{g}}_3{\mathrm{g}}_2{\mathrm{g}}_1\right){\Phi}_{\left(\mathrm{N}\right)}=\left[\left({\mathrm{g}}_{\left(\mathrm{n}\hbox{-} 1\right)}\dots {\mathrm{g}}_3{\mathrm{g}}_2{\mathrm{g}}_1\right){\upvarphi}_{(1)\mathrm{m}},\left({\mathrm{g}}_{\left(\mathrm{n}\hbox{-} 1\right)}\dots {\mathrm{g}}_3{\mathrm{g}}_2{\mathrm{g}}_1\right){\upvarphi}_{(2)\mathrm{m}},\dots, \left({\mathrm{g}}_{\left(\mathrm{n}\hbox{-} 1\right)}\dots {\mathrm{g}}_3{\mathrm{g}}_2{\mathrm{g}}_1\right){\upvarphi}_{\left(\upnu \right)\mathrm{m}},\dots, \left({\mathrm{g}}_{\left(\mathrm{n}\hbox{-} 1\right)}\dots {\mathrm{g}}_3{\mathrm{g}}_2{\mathrm{g}}_1\right)\ {\upvarphi}_{\left(\mathrm{N}\right)\mathrm{m}}\right]\\ {} = \left[{\upvarphi}_{(1)\mathrm{m}},\ {\upvarphi}_{(2)\mathrm{m}},\dots,\ {\upvarphi}_{\left(\upnu \right)\mathrm{m}},\dots,\ {\upvarphi}_{\left(\mathrm{N}\right)\mathrm{m}}\right]\left[{}_{\mathrm{m}}{}^{\mathrm{N}}\mathrm{D}\left({\mathrm{g}}_{\left(\mathrm{n}\hbox{-} 1\right)}\right)\right]\dots \left[{}_{\mathrm{m}}{}^{\mathrm{N}}\mathrm{D}\left({\mathrm{g}}_3\right)\right]\left[{}_{\mathrm{m}}{}^{\mathrm{N}}\mathrm{D}\left({\mathrm{g}}_2\right)\right]\left[{}_{\mathrm{m}}{}^{\mathrm{N}}\mathrm{D}\left({\mathrm{g}}_1\right)\right],\end{array} $$

$$ = {\Phi}_{\left(\mathrm{N}\right)}\left[{}_{\mathrm{m}}{}^{\mathrm{N}}\mathrm{D}\left({\mathrm{g}}_{\left(\mathrm{n}\hbox{-} 1\right)}\right)\right]\dots \left[{}_{\mathrm{m}}{}^{\mathrm{N}}\mathrm{D}\left({\mathrm{g}}_3\right)\right]\left[{}_{\mathrm{m}}{}^{\mathrm{N}}\mathrm{D}\left({\mathrm{g}}_2\right)\right]\left[{}_{\mathrm{m}}{}^{\mathrm{N}}\mathrm{D}\left({\mathrm{g}}_1\right)\right]. $$

Alternatively, operating (37) and (38) on the coordinates gives φ_(ν)m_[_m_^N^D(g)]_ν_ = [_m_^N^D(g)]_ν_ in the *v*-th layer. Moreover, when *μ* ≠ ν, (##) ensures that

45 $$ {\upvarphi}_{\left(\upmu \right)\mathrm{m}}{\left[{}_{\mathrm{m}}^{\mathrm{N}}\mathrm{D}\left(\mathrm{g}\right)\right]}_{\upmu}=0\kern0.5em \left(\mathrm{an}\kern0.5em m\kern0.5em \times \kern0.5em m\kern0.5em \mathrm{zeromatrix}\right). $$

Note that (44) is a simple combination of products; that is, (g_(n ‐ 1)_ … g_3_g_2_g_1_)φ_(ν)m_ = φ_(ν)m_[_m_^N^D(g)]_ν_ in all layers *v* = 1, 2,…, N.

Using these definitions, we can represent combinations of arbitrarily selected R^m^s (20) or R^m'^s (23) from M in similar matrix form.

#### §3. Practical demonstration using simple examples

To facilitate understanding of our approach, we apply (20)–(44) to some simple practical examples.

Suppose that R^m^s are arbitrarily selected from (20) and labeled R_(0→1)_, R_(1→2)_, and R_(2→3)_ (i.e., *m* = 3, denoting 3 assessment sessions). Consider that R^m'^s (R_(0 → 1)_^'^, R_(1 → 2)_^'^ and R_(2 → 3)_^'^) are selected from the same patient, or can be combined for arbitrary categorization purposes (e.g., R_a_, R_b_, R_c_, R_d_,…). For an easy understanding, we presume that R^m^ and R^m'^ are the clinical courses of a certain patient without and with intervention/treatment T, respectively, over the entire admission. We can define a mapping τ such as (20), (23), (25) and (26). Obviously, the courses R^m^ and R^m'^ cannot run simultaneously; however, we presume that after a sufficient scientific investigation of the intervention T, we can determine the most expected interaction among any components of the matrices _3_^N^D (with and without T) for a given patient. Therefore, we can envisage the following scenario.

If the number of layers is 4 (*N* = 4), the plural sets of the patients’ disease states, given R^m^ without intervention T and R^m'^ with T, can be determined through the mapping τ (20):

46 $$ \uptau :\kern0.5em {\mathrm{R}}^3 \equiv \left[\begin{array}{c}\hfill \mathrm{R}\left(0\to 1\right)\hfill \\ {}\hfill \mathrm{R}\left(1\to 2\right)\hfill \\ {}\hfill \mathrm{R}\left(2\to 3\right)\hfill \end{array}\right]\left(3\times 1\ \mathrm{column}\ \mathrm{matrix}\right)\ \to {{\mathrm{R}}^3}^{\hbox{'}}\equiv \left[\begin{array}{c}\hfill \mathrm{R}{\left(0\to 1\right)}^{\hbox{'}}\hfill \\ {}\hfill \mathrm{R}{\left(1\to 2\right)}^{\hbox{'}}\hfill \\ {}\hfill \mathrm{R}{\left(2\to 3\right)}^{\hbox{'}}\hfill \end{array}\right]\left(3\times 1\ \mathrm{column}\ \mathrm{matrix}\right). $$

Then, during the clinical course R^m'^ of a patient undergoing T, the individual states at each session are expressed as R_(0 → 1)'_, R_(1 → 2)'_, R_(2 → 3)'_.

From (25), for g (∈ GL(V^m^)), we have

47 $$ {\mathrm{gR}}^3{{=\mathrm{R}}^3}^{\hbox{'}}\left(\mathrm{m}\kern0.5em  = 3\right). $$

According to (25) and (26),

48 $$ {{\mathrm{R}}^3}^{\hbox{'}}=\left[{}_3^{\mathrm{N}}\mathrm{D}\left(\mathrm{g}\right)\right]{\mathrm{R}}^3, $$

or in the *v*-th layer (*v* = 1,2,…,N),

49 $$ {\mathrm{R}}_{\upnu}^{3\hbox{'}}={\left[{}_3^{\mathrm{N}}\mathrm{D}\left(\mathrm{g}\right)\right]}_{\upnu}{\mathrm{R}}_{\upnu}^3, $$

50 $$ {\mathrm{R}}_{\upnu}^{3\hbox{'}}=\left[\begin{array}{ccc}\hfill {\mathrm{D}}_{\left(\upnu \right)11}\hfill & \hfill {\mathrm{D}}_{\left(\upnu \right)12}\hfill & \hfill {\mathrm{D}}_{\left(\upnu \right)13}\hfill \\ {}\hfill {\mathrm{D}}_{\left(\upnu \right)21}\hfill & \hfill {\mathrm{D}}_{\left(\upnu \right)22}\hfill & \hfill {\mathrm{D}}_{\left(\upnu \right)23}\hfill \\ {}\hfill {\mathrm{D}}_{\left(\upnu \right)31}\hfill & \hfill {\mathrm{D}}_{\left(\upnu \right)32}\hfill & \hfill {\mathrm{D}}_{\left(\upnu \right)33}\hfill \end{array}\right]{\mathrm{R}}_{\upnu}^3. $$

For instance, an arbitrary R^3^ is given by

51 $$ {\mathrm{R}}_{\left(0\to 1\right)}=\left[\begin{array}{c}\hfill \left\{10\kern1em \left( \mod \kern0.5em 11\right)\right\}/10\hfill \\ {}\hfill \left\{6\kern1.12em \left( \mod \kern0.5em 7\right)\right\}/6\hfill \\ {}\hfill 148\kern0.5em \left(\mathrm{mg}/\mathrm{dL}\right)\kern1em \hfill \\ {}\hfill 6\kern0.5em \left(1/24\mathrm{hrs}\right)\hfill \end{array}\right],\kern0.5em {\mathrm{R}}_{\left(1\to 2\right)}=\left[\begin{array}{c}\hfill \left\{-3\kern1em \left( \mod \kern0.5em 11\right)\right\}/10\hfill \\ {}\hfill \left\{-2\kern1.12em \left( \mod \kern0.5em 7\right)\right\}/6\hfill \\ {}\hfill -22\kern0.5em \left(\mathrm{mg}/\mathrm{dL}\right)\kern1em \hfill \\ {}\hfill 1\kern0.5em \left(1/24\mathrm{hrs}\right)\hfill \end{array}\right],\kern0.5em {\mathrm{R}}_{\left(2\to 3\right)}=\left[\begin{array}{c}\hfill \left\{2\kern1em \left( \mod \kern0.5em 11\right)\right\}/10\hfill \\ {}\hfill \left\{1\kern1.12em \left( \mod \kern0.5em 7\right)\right\}/6\hfill \\ {}\hfill 15\kern0.5em \left(\mathrm{mg}/\mathrm{dL}\right)\kern1em \hfill \\ {}\hfill -3\kern0.5em \left(1/24\mathrm{hrs}\right)\hfill \end{array}\right]. $$

Note that to meaningfully express the modular score as a ratio, we divide the first components (first layer) by 10 (=‘p − 1’ at mod p, with *p* = 11), and interpret the severity of the first symptom on a 10-point scale {0, 1/10, 2/10,…,10/10} = Z_11_/10. Similarly, we divide the second component (second layer) by 6 (=7 − 1), and interpret its severity on a 6-point scale {0, 1/6, 2/6,…,6/6} = Z_7_/6.

The representation matrix $\left[{}_3^{\mathrm{N}}\mathrm{D}\left(\mathrm{g}\right)\right]$ of the patient after sufficient investigation is given by

52 $$ \begin{array}{l}{\left[{}_3{}^4\mathrm{D}\left(\mathrm{g}\right)\right]}_1 = \left[\begin{array}{ccc}\hfill 1\hfill & \hfill 3\hfill & \hfill 4\hfill \\ {}\hfill 0\hfill & \hfill 2\hfill & \hfill 5\hfill \\ {}\hfill 1\hfill & \hfill 4\hfill & \hfill 2\hfill \end{array}\right]\kern0.5em \left( \mod \kern0.5em 11\right),\ {\left[{}_3{}^4\mathrm{D}\left(\mathrm{g}\right)\right]}_2 = \left[\begin{array}{ccc}\hfill 1\hfill & \hfill 2\hfill & \hfill 3\hfill \\ {}\hfill 0\hfill & \hfill 3\hfill & \hfill 4\hfill \\ {}\hfill 1\hfill & \hfill 2\hfill & \hfill 1\hfill \end{array}\right]\kern0.5em \left( \mod \kern0.5em 7\right),\\ {}{\left[{}_3{}^4\mathrm{D}\left(\mathrm{g}\right)\right]}_3 = \left[\begin{array}{ccc}\hfill 0.9\hfill & \hfill 0.2\hfill & \hfill 0.3\hfill \\ {}\hfill 0.5\hfill & \hfill 1.2\hfill & \hfill -0.7\hfill \\ {}\hfill 0.4\hfill & \hfill -0.2\hfill & \hfill 0.6\hfill \end{array}\right]\left(\mathrm{mg}/\mathrm{dL}\right),\ {\left[{}_3{}^4\mathrm{D}\left(\mathrm{g}\right)\right]}_4 = \left[\begin{array}{ccc}\hfill 1.1\hfill & \hfill 0.2\hfill & \hfill -0.1\hfill \\ {}\hfill 0.3\hfill & \hfill -0.5\hfill & \hfill 0.7\hfill \\ {}\hfill 0.1\hfill & \hfill 0.7\hfill & \hfill 0.3\hfill \end{array}\right]\left(1/24\mathrm{hrs}\right).\end{array} $$

In detail, supposing that ◊ expresses mod 11 addition in the first layer, we can write,

53 $$ \left(1/10\right)\ \left\{\left[\begin{array}{ccc}\hfill 1\hfill & \hfill 3\hfill & \hfill 4\hfill \\ {}\hfill 0\hfill & \hfill 2\hfill & \hfill 5\hfill \\ {}\hfill 1\hfill & \hfill 4\hfill & \hfill 2\hfill \end{array}\right]\kern0.5em \left[\begin{array}{c}\hfill 10\hfill \\ {}\hfill \lozenge \hfill \\ {}\hfill -3\hfill \\ {}\hfill \lozenge \hfill \\ {}\hfill 2\hfill \\ {}\hfill \left(\Rightarrow 9\right)\hfill \end{array}\right]\left( \mod \kern0.5em 11\right)\right\} = \left(1/10\right)\left\{\left[\begin{array}{c}\hfill 9\hfill \\ {}\hfill \lozenge \hfill \\ {}\hfill 4\hfill \\ {}\hfill \lozenge \hfill \\ {}\hfill 2\kern0.5em \left(=-9\right)\hfill \\ {}\hfill \left(\Rightarrow 4\right)\hfill \end{array}\right]\left( \mod \kern0.5em 11\right)\right\}. $$

Similarly, for the second layer,

54 $$ \left(1/6\right)\left\{\left[\begin{array}{ccc}\hfill 1\hfill & \hfill 2\hfill & \hfill 3\hfill \\ {}\hfill 0\hfill & \hfill 3\hfill & \hfill 4\hfill \\ {}\hfill 1\hfill & \hfill 2\hfill & \hfill 1\hfill \end{array}\right]\kern0.5em \left[\begin{array}{c}\hfill 6\hfill \\ {}\hfill \lozenge \hfill \\ {}\hfill -2\hfill \\ {}\hfill \lozenge \hfill \\ {}\hfill 1\hfill \\ {}\hfill \left(\Rightarrow 5\right)\hfill \end{array}\right]\left( \mod \kern0.5em 7\right)\right\}\kern0.75em  = \left(1/6\right)\left\{\left[\begin{array}{c}\hfill 5\hfill \\ {}\hfill \lozenge \hfill \\ {}\hfill -2\hfill \\ {}\hfill \lozenge \hfill \\ {}\hfill 3\hfill \\ {}\hfill \left(\Rightarrow 6\right)\hfill \end{array}\right]\left( \mod \kern0.5em 7\right)\right\}. $$

For the third layer, we have

55 $$ \left[\begin{array}{ccc}\hfill 0.9\hfill & \hfill 0.2\hfill & \hfill 0.3\hfill \\ {}\hfill 0.5\hfill & \hfill 1.2\hfill & \hfill -0.7\hfill \\ {}\hfill 0.4\hfill & \hfill -0.2\hfill & \hfill 0.6\hfill \end{array}\right]\left[\begin{array}{c}\hfill 148\hfill \\ {}\hfill \lozenge \hfill \\ {}\hfill -22\hfill \\ {}\hfill \lozenge \hfill \\ {}\hfill 15\hfill \\ {}\hfill \left(\Rightarrow 141\right)\hfill \end{array}\right]\left(\mathrm{mg}/\mathrm{dL}\right) = \left[\begin{array}{c}\hfill 133.3\hfill \\ {}\hfill \lozenge \hfill \\ {}\hfill 37.1\hfill \\ {}\hfill \lozenge \hfill \\ {}\hfill 72.6\hfill \\ {}\hfill \left(\Rightarrow 243\right)\hfill \end{array}\right]\left(\mathrm{mg}/\mathrm{dL}\right). $$

Finally, for the fourth layer, we have

56 $$ \left[\begin{array}{ccc}\hfill 1.1\hfill & \hfill 0.2\hfill & \hfill -0.1\hfill \\ {}\hfill 0.3\hfill & \hfill -0.5\hfill & \hfill 0.7\hfill \\ {}\hfill 0.1\hfill & \hfill 0.7\hfill & \hfill 0.3\hfill \end{array}\right]\left[\begin{array}{c}\hfill 6\hfill \\ {}\hfill \lozenge \hfill \\ {}\hfill 1\hfill \\ {}\hfill \lozenge \hfill \\ {}\hfill -3\hfill \\ {}\hfill \left(\Rightarrow 4\right)\hfill \end{array}\right]\left(1/24\mathrm{hrs}\right) = \kern0.5em \left[\begin{array}{c}\hfill 7.1\hfill \\ {}\hfill \lozenge \hfill \\ {}\hfill -0.8\hfill \\ {}\hfill \lozenge \hfill \\ {}\hfill 0.4\hfill \\ {}\hfill \left(\Rightarrow 6.7\right)\hfill \end{array}\right]\left(1/24\mathrm{hrs}\right). $$

Ordinarily, the non-modular elements in matrices are unit-less numbers; therefore, the units mg/dL and 1/24 h are meaningless in the above matrix calculations. Here they are included for analogy with our operation ◊, as in [19]. If ◊ is merely regarded as a mark, we can define operations that are incompatible with standard matrix operations, such as non-mod/mod p_ν_ multiplication or division (p_ν_: prime).

Equations (48)–(52) uniquely define R^3'^:

57 $$ {\mathrm{R}}_{{\left(0\to 1\right)}^{\hbox{'}}}=\left[\begin{array}{c}\hfill \left\{9\kern1em \left( \mod \kern0.5em 11\right)\right\}/10\hfill \\ {}\hfill \left\{5\kern1.12em \left( \mod \kern0.5em 7\right)\right\}/6\hfill \\ {}\hfill 133.3\kern0.5em \left(\mathrm{mg}/\mathrm{dL}\right)\kern1em \hfill \\ {}\hfill 7.1\kern0.5em \left(1/24\mathrm{hrs}\right)\hfill \end{array}\right],\kern0.5em {\mathrm{R}}_{{\left(1\to 2\right)}^{\hbox{'}}}=\left[\begin{array}{c}\hfill \left\{4\kern1em \left( \mod \kern0.5em 11\right)\right\}/10\hfill \\ {}\hfill \left\{-2\kern1.12em \left( \mod \kern0.5em 7\right)\right\}/6\hfill \\ {}\hfill 37.1\kern0.5em \left(\mathrm{mg}/\mathrm{dL}\right)\kern1em \hfill \\ {}\hfill -0.8\kern0.5em \left(1/24\mathrm{hrs}\right)\hfill \end{array}\right],\kern0.5em {\mathrm{R}}_{{\left(2\to 3\right)}^{\hbox{'}}}=\left[\begin{array}{c}\hfill \left\{2\kern1em \left( \mod \kern0.5em 11\right)\right\}/10\hfill \\ {}\hfill \left\{3\kern1.12em \left( \mod \kern0.5em 7\right)\right\}/6\hfill \\ {}\hfill 72.6\kern0.5em \left(\mathrm{mg}/\mathrm{dL}\right)\kern1em \hfill \\ {}\hfill 0.4\kern0.5em \left(1/24\mathrm{hrs}\right)\hfill \end{array}\right]. $$

For R_(j-1→j)_ in (51) or R_(j − 1 → j)'_ in (53) (*j* = 1, 2, 3), we presume the following scenario; the first row (layer) in (51) or (53) describes the severity of a patient’s anemia (or whether the severity increases or decreases) via modulo 11 addition: 0 implies no symptoms/clinical findings, 10 denotes maximally severe anemia, and 5 is the medium severity of anemia. Here, all of the numbers are divided by 10. Similarly, the second row (layer) expresses the severity of the patient’s depression via modulo 7 addition; hence, all numbers are divided by 6. The third row (layer) expresses the patient’s fasting blood sugar (FBS) level (mg/dL) recorded in the morning, and the fourth row (layer) expresses the sleep time per day (1/24 h). The third and fourth layers involve an ordinal addition. For instance, in the fourth layer, the inverse of an element x (a patient’s sleep time) is x^−1^ = 24 − x (1/24 h). As a simple illustration, let R_(0 → 1)'_ be the disease state of a certain patient on day 1 of treatment, and R_(1 → 2)'_ and R_(2 → 3)'_ represent the changes between days 1 and 2 and between days 2 and 3, respectively. In terms of (23), the optional expression (21) can be calculated as

58 $$ \left[{\mathrm{R}}_{\left(0\to 1\right)}\lozenge {{\mathrm{R}}_{\Big(1}}_{\to 2\Big)}\lozenge {{\mathrm{R}}_{\Big(2}}_{\to 3\Big)}\left(={\mathrm{R}}_3\right)\right]. $$

Although the operator ◊ admits non-modulo/modulo arbitrary arithmetic, we here choose ‘◊ = * (addition)’ for simplicity, and present the calculations as row vectors for easy visualization. Then, from (51), we have

59 $$ \begin{array}{l}{\mathrm{R}}_{\left(0\to 1\right)}\lozenge {{\mathrm{R}}_{\Big(1}}_{\to 2\Big)}\lozenge {{\mathrm{R}}_{\Big(2}}_{\to 3\Big)} = {\mathrm{R}}_{\left(0\to 1\right)}{{+\mathrm{R}}_{\Big(1}}_{\to 2\Big)}{{+\mathrm{R}}_{\Big(2}}_{\to 3\Big)} = \left[\left\{10\ \left( \mod\ 11\right)\right\}/{10}_1\left|\ \left\{6\ \left( \mod\ 7\right)\right\}/{6}_2\right|\ 148\ {\left(\mathrm{mg}/\mathrm{dL}\right)}_3\Big|\ 6\ {\left(1/24\mathrm{hrs}\right)}_4\right]\\ {} + \left[\left\{-3\ \left( \mod\ 11\right)\right\}/{10}_1\left|\ \left\{-2\ \left( \mod\ 7\right)\right\}/{6}_2\right| - 22\ {\left(\mathrm{mg}/\mathrm{dL}\right)}_3\Big|\ 1\ {\left(1/24\mathrm{hrs}\right)}_4\right] + \Big[\left\{2\ \left( \mod\ 11\right)\right\}/{10}_1\left|\ \left\{1\ \left( \mod\ 7\right)\right\}/{6}_2\right|\ 15\ {\left(\mathrm{mg}/\mathrm{dL}\right)}_3\Big|\\ {}-3\ {\left(1/24\mathrm{hrs}\right)}_4\left] = \right[\left\{10-3+2\ \left( \mod\ 11\right)\right\}/{10}_1\left|\ \left\{6-2+1\ \left( \mod\ 7\right)\right\}/{6}_2\right|\ 148-22+15\ {\left(\mathrm{mg}/\mathrm{dL}\right)}_3\left|\ 6+1-3\ {\left(1/24\mathrm{hrs}\right)}_4\right]\\ {} = \left[\left\{9\ \left( \mod\ 11\right)\right\}/{10}_1\left|\ \left\{5\ \left( \mod\ 7\right)\right\}/{6}_2\right|\ 141\ {\left(\mathrm{mg}/\mathrm{dL}\right)}_3\Big|\ 4\ {\left(1/24\mathrm{hrs}\right)}_4\right] = {\mathrm{R}}_3.\end{array} $$

Here, the indexes indicate the number of layers.

As is seen, optionally, to explicitly define the series of operations (58), we could express (46) and (48) in terms of ◊, preserving the ordinal meaning of column vectors (without ◊):

60 $$ \uptau :\kern0.5em {\mathrm{R}}^3\equiv \left[\begin{array}{c}\hfill \mathrm{R}\left(0\to 1\right)\hfill \\ {}\hfill \lozenge \hfill \\ {}\hfill \mathrm{R}\left(1\to 2\right)\hfill \\ {}\hfill \lozenge \hfill \\ {}\hfill \mathrm{R}\left(2\to 3\right)\hfill \end{array}\right]\to {{\mathrm{R}}^3}^{\hbox{'}}\kern0.5em \equiv \left[\begin{array}{c}\hfill \mathrm{R}{\left(0\to 1\right)}^{\hbox{'}}\hfill \\ {}\hfill \lozenge \hfill \\ {}\hfill \mathrm{R}{\left(1\to 2\right)}^{\hbox{'}}\hfill \\ {}\hfill \lozenge \hfill \\ {}\hfill \mathrm{R}{\left(2\to 3\right)}^{\hbox{'}}\hfill \end{array}\right], $$

61 $$ \left[\begin{array}{c}\hfill \mathrm{R}{\left(0\to 1\right)}^{\hbox{'}}\hfill \\ {}\hfill \lozenge \hfill \\ {}\hfill \mathrm{R}{\left(1\to 2\right)}^{\hbox{'}}\hfill \\ {}\hfill \lozenge \hfill \\ {}\hfill \mathrm{R}{\left(2\to 3\right)}^{\hbox{'}}\hfill \end{array}\right]=\left[{}_3^{\mathrm{N}}\mathrm{D}\left(\mathrm{g}\right)\right]\left[\begin{array}{c}\hfill \mathrm{R}\left(0\to 1\right)\hfill \\ {}\hfill \lozenge \hfill \\ {}\hfill \mathrm{R}\left(1\to 2\right)\hfill \\ {}\hfill \lozenge \hfill \\ {}\hfill \mathrm{R}\left(2\to 3\right)\hfill \end{array}\right]. $$

Additionally, in (61), the following relationship is naturally confirmable if the rules of ◊ are determined according to the right-hand equation of (28) in the same order:

62 $$ \begin{array}{l}\mathrm{R}{\left(0\to 1\right)}^{\hbox{'}}\lozenge \mathrm{R}{\left(1\to 2\right)}^{\hbox{'}}\lozenge \mathrm{R}{\left(2\to 3\right)}^{\hbox{'}}=\mathrm{R}{\left(0\to 1\right)}^{\hbox{'}}+\mathrm{R}{\left(1\to 2\right)}^{\hbox{'}}+\mathrm{R}{\left(2\to 3\right)}^{\hbox{'}}\\ {}=\left[\left\{9+4+2\ \left( \mod\ 11\right)\right\}/{10}_1\Big|\ \left\{5-2+3\ \left( \mod\ 7\right)\right\}/{6}_2\left|\ 133.3+37.1+72.6\ {\left(\mathrm{mg}/\mathrm{dL}\right)}_3\right|\ 7.1-0.8+0.4\ {\left(1/24\mathrm{hrs}\right)}_4\right]\\ {}=\left[\left\{4\ \left( \mod\ 11\right)\right\}/{10}_1\Big|\ \left\{6\left( \mod\ 7\right)\right\}/{6}_2\left|\ 243.0\ {\left(\mathrm{mg}/\mathrm{dL}\right)}_3\right|\ 6.7\ {\left(1/24\mathrm{hrs}\right)}_4\right]={\mathrm{R}}_3^{\hbox{'}}.\end{array} $$

If these conditions fail, ◊ might be inconsistently defined on both sides of equation (28), causing confusion.

In (50), the conditions (51) and (57) are insufficient to uniquely determine a matrix such as (52). Conversely, if the solutions (51) and (52) are obtained beforehand, based on additional conditions provided by specific postulates, various cases are permitted.

The representation matrices $\left[{}_3^{\mathrm{N}}\mathrm{D}\left(\mathrm{g}\right)\right]$ in (49) are combinations of the matrices in (52) from the first to the fourth layers in ascending order from bottom to top: ${\left[{}_3^4\mathrm{D}\left(\mathrm{g}\right)\right]}_1,{\left[{}_3^4\mathrm{D}\left(\mathrm{g}\right)\right]}_2,{\left[{}_3^4\mathrm{D}\left(\mathrm{g}\right)\right]}_3,{\left[{}_3^4\mathrm{D}\left(\mathrm{g}\right)\right]}_4.$

Furthermore, as a more complicated demonstration of (33) and (34), we re-denote the above operation g (intervention T) by g_1_, and introduce two new operations g_2_ and g_3_, representing a drug A that reduces FBS (mg/dL) and a therapy B that improves anemia, respectively.

Under the following matrix representations of g_2_ (drug A), R^3'^ transforms into

63 $$ {{\mathrm{R}}^3}^{\hbox{'}\hbox{'}}=\left[{}_3^{\mathrm{N}}\mathrm{D}\left({\mathrm{g}}_2\right)\right]{{\mathrm{R}}^3}^{\hbox{'}}. $$

64 $$ \begin{array}{l}{\left[{}_3{}^4\mathrm{D}\left(\mathrm{g}\right)\right]}_1=\left[\begin{array}{ccc}\hfill 1\hfill & \hfill 0\hfill & \hfill 0\hfill \\ {}\hfill 0\hfill & \hfill 1\hfill & \hfill 0\hfill \\ {}\hfill 0\hfill & \hfill 0\hfill & \hfill 1\hfill \end{array}\right]\kern0.5em \left( \mod \kern0.5em 11\right),\kern0.5em {\left[{}_3{}^4\mathrm{D}\left(\mathrm{g}\right)\right]}_2=\left[\begin{array}{ccc}\hfill 1\hfill & \hfill 0\hfill & \hfill 0\hfill \\ {}\hfill 0\hfill & \hfill 1\hfill & \hfill 0\hfill \\ {}\hfill 0\hfill & \hfill 0\hfill & \hfill 1\hfill \end{array}\right]\kern0.5em \left( \mod \kern0.5em 7\right),\\ {}{\left[{}_3{}^4\mathrm{D}\left(\mathrm{g}\right)\right]}_3=\left[\begin{array}{ccc}\hfill 0.9\hfill & \hfill 0\hfill & \hfill 0\hfill \\ {}\hfill 0\hfill & \hfill -0.8\hfill & \hfill 0\hfill \\ {}\hfill 0\hfill & \hfill 0\hfill & \hfill -0.3\hfill \end{array}\right]\left(\mathrm{mg}/\mathrm{dL}\right),\kern0.75em {\left[{}_3{}^4\mathrm{D}\left(\mathrm{g}\right)\right]}_4=\left[\begin{array}{ccc}\hfill 1\hfill & \hfill 0\hfill & \hfill 0\hfill \\ {}\hfill 0\hfill & \hfill 1\hfill & \hfill 0\hfill \\ {}\hfill 0\hfill & \hfill 0\hfill & \hfill 1\hfill \end{array}\right]\left(1/24\mathrm{hrs}\right).\end{array} $$

For simplicity, we assume that drug A affects only the third component (layer):

$$ \left[\begin{array}{ccc}\hfill 0.9\hfill & \hfill 0\hfill & \hfill 0\hfill \\ {}\hfill 0\hfill & \hfill -0.8\hfill & \hfill 0\hfill \\ {}\hfill 0\hfill & \hfill 0\hfill & \hfill -0.3\hfill \end{array}\right]\left[\begin{array}{c}\hfill 133.3\hfill \\ {}\hfill \lozenge \hfill \\ {}\hfill 37.1\hfill \\ {}\hfill \lozenge \hfill \\ {}\hfill 72.6\hfill \\ {}\hfill \left(\Rightarrow 243\right)\hfill \end{array}\right]\left(\mathrm{mg}/\mathrm{dL}\right) = \left[\begin{array}{c}\hfill 120.0\hfill \\ {}\hfill \lozenge \hfill \\ {}\hfill 29.7\hfill \\ {}\hfill \lozenge \hfill \\ {}\hfill 21.8\hfill \\ {}\hfill \left(\Rightarrow 171.5\right)\hfill \end{array}\right]\left(\mathrm{mg}/\mathrm{dL}\right). $$

Thereby, according to (63) and (64),

R^3' '^ is obtained as follows:

65 $$ {\mathrm{R}}_{{\left(0\to 1\right)}^{\hbox{'}\hbox{'}}}=\left[\begin{array}{c}\hfill \left\{9\kern1em \left( \mod \kern0.5em 11\right)\right\}/10\hfill \\ {}\hfill \left\{5\kern1.12em \left( \mod \kern0.5em 7\right)\right\}/6\hfill \\ {}\hfill 120.0\kern0.5em \left(\mathrm{mg}/\mathrm{dL}\right)\kern1em \hfill \\ {}\hfill 7.1\kern0.5em \left(1/24\mathrm{hrs}\right)\hfill \end{array}\right],\kern0.5em {\mathrm{R}}_{{\left(1\to 2\right)}^{\hbox{'}\hbox{'}}}=\left[\begin{array}{c}\hfill \left\{4\kern1em \left( \mod \kern0.5em 11\right)\right\}/10\hfill \\ {}\hfill \left\{-2\kern1.12em \left( \mod \kern0.5em 7\right)\right\}/6\hfill \\ {}\hfill 29.7\kern0.5em \left(\mathrm{mg}/\mathrm{dL}\right)\kern1em \hfill \\ {}\hfill -0.8\kern0.5em \left(1/24\mathrm{hrs}\right)\hfill \end{array}\right],\kern0.5em {\mathrm{R}}_{{\left(2\to 3\right)}^{\hbox{'}\hbox{'}}}=\left[\begin{array}{c}\hfill \left\{2\kern1em \left( \mod \kern0.5em 11\right)\right\}/10\hfill \\ {}\hfill \left\{3\kern1.12em \left( \mod \kern0.5em 7\right)\right\}/6\hfill \\ {}\hfill 21.8\kern0.5em \left(\mathrm{mg}/\mathrm{dL}\right)\kern1em \hfill \\ {}\hfill 0.4\kern0.5em \left(1/24\mathrm{hrs}\right)\hfill \end{array}\right]. $$

Additionally, under the following matrix representations of g_3_ (therapy B), R^3' '^ transforms into

66 $$ {{\mathrm{R}}^3}^{\hbox{'}\hbox{'}\hbox{'}}=\left[{}_3^{\mathrm{N}}\mathrm{D}\left({\mathrm{g}}_2\right)\right]{{\mathrm{R}}^3}^{\hbox{'}\hbox{'}}. $$

67 $$ \begin{array}{l}{\left[{}_3{}^4\mathrm{D}\left(\mathrm{g}\right)\right]}_1=\left[\begin{array}{ccc}\hfill 1\hfill & \hfill 0\hfill & \hfill 0\hfill \\ {}\hfill 0\hfill & \hfill -2\hfill & \hfill 0\hfill \\ {}\hfill 0\hfill & \hfill 0\hfill & \hfill 1\hfill \end{array}\right]\kern0.5em \left( \mod \kern0.5em 11\right),\kern0.5em {\left[{}_3{}^4\mathrm{D}\left(\mathrm{g}\right)\right]}_2=\left[\begin{array}{ccc}\hfill 1\hfill & \hfill 0\hfill & \hfill 0\hfill \\ {}\hfill 0\hfill & \hfill 1\hfill & \hfill 0\hfill \\ {}\hfill 0\hfill & \hfill 0\hfill & \hfill 1\hfill \end{array}\right]\kern0.5em \left( \mod \kern0.5em 7\right),\\ {}{\left[{}_3{}^4\mathrm{D}\left(\mathrm{g}\right)\right]}_3=\left[\begin{array}{ccc}\hfill 1\hfill & \hfill 0\hfill & \hfill 0\hfill \\ {}\hfill 0\hfill & \hfill 1\hfill & \hfill 0\hfill \\ {}\hfill 0\hfill & \hfill 0\hfill & \hfill 1\hfill \end{array}\right]\left(\mathrm{mg}/\mathrm{dL}\right),\kern0.5em {\left[{}_3{}^4\mathrm{D}\left(\mathrm{g}\right)\right]}_4=\left[\begin{array}{ccc}\hfill 1\hfill & \hfill 0\hfill & \hfill 0\hfill \\ {}\hfill 0\hfill & \hfill 1\hfill & \hfill 0\hfill \\ {}\hfill 0\hfill & \hfill 0\hfill & \hfill 1\hfill \end{array}\right]\left(1/24\mathrm{hrs}\right).\end{array} $$

Again for simplicity, we assume that therapy B affects only the first component (layer):

$$ \begin{array}{l}\left(1/10\right)\left\{\left[\begin{array}{ccc}\hfill 1\hfill & \hfill 0\hfill & \hfill 0\hfill \\ {}\hfill 0\hfill & \hfill -2\hfill & \hfill 0\hfill \\ {}\hfill 0\hfill & \hfill 0\hfill & \hfill 1\hfill \end{array}\right]\kern0.5em \left( \mod \kern0.5em 11\right)\right\}\left\{\left[\begin{array}{c}\hfill 9\hfill \\ {}\hfill \lozenge \hfill \\ {}\hfill 4\hfill \\ {}\hfill \lozenge \hfill \\ {}\hfill 2\kern0.5em \left(=-9\right)\hfill \\ {}\hfill \left(\Rightarrow 4\right)\hfill \end{array}\right]\left( \mod \kern0.5em 11\right)\right\}\\ {} = \left(1/10\right)\left\{\left[\begin{array}{ccc}\hfill 1\hfill & \hfill 0\hfill & \hfill 0\hfill \\ {}\hfill 0\hfill & \hfill -2\hfill & \hfill 0\hfill \\ {}\hfill 0\hfill & \hfill 0\hfill & \hfill 1\hfill \end{array}\right]\kern0.5em \left[\begin{array}{c}\hfill 9\hfill \\ {}\hfill \lozenge \hfill \\ {}\hfill 4\hfill \\ {}\hfill \lozenge \hfill \\ {}\hfill 2\kern0.5em \left(=-9\right)\hfill \\ {}\hfill \left(\Rightarrow 4\right)\hfill \end{array}\right]\left( \mod \kern0.5em 11\right)\right\} = \left(1/10\right)\left\{\left[\begin{array}{c}\hfill 9\hfill \\ {}\hfill \lozenge \hfill \\ {}\hfill -8\hfill \\ {}\hfill \lozenge \hfill \\ {}\hfill 2\hfill \\ {}\hfill \left(\Rightarrow 3\right)\hfill \end{array}\right]\left( \mod \kern0.5em 11\right)\right\}\\ {} = \left(1/10\right)\left\{\left[\begin{array}{c}\hfill 9\hfill \\ {}\hfill \lozenge \hfill \\ {}\hfill 3\hfill \\ {}\hfill \lozenge \hfill \\ {}\hfill 2\hfill \\ {}\hfill \left(\Rightarrow 3\right)\hfill \end{array}\right]\left( \mod \kern0.5em 11\right)\right\}\end{array} $$

Using (66) and (67),

R^3' ' '^ is then calculated as follows:

68 $$ {\mathrm{R}}_{{\left(0\to 1\right)}^{\hbox{'}\hbox{'}\hbox{'}}}=\left[\begin{array}{c}\hfill \left\{9\kern1em \left( \mod \kern0.5em 11\right)\right\}/10\hfill \\ {}\hfill \left\{5\kern1.12em \left( \mod \kern0.5em 7\right)\right\}/6\hfill \\ {}\hfill 120.0\kern0.5em \left(\mathrm{mg}/\mathrm{dL}\right)\kern1em \hfill \\ {}\hfill 7.1\kern0.5em \left(1/24\mathrm{hrs}\right)\hfill \end{array}\right],\kern0.5em {\mathrm{R}}_{{\left(1\to 2\right)}^{\hbox{'}\hbox{'}\hbox{'}}}=\left[\begin{array}{c}\hfill \left\{3\kern1em \left( \mod \kern0.5em 11\right)\right\}/10\hfill \\ {}\hfill \left\{-2\kern1.12em \left( \mod \kern0.5em 7\right)\right\}/6\hfill \\ {}\hfill 29.7\kern0.5em \left(\mathrm{mg}/\mathrm{dL}\right)\kern1em \hfill \\ {}\hfill -0.8\kern0.5em \left(1/24\mathrm{hrs}\right)\hfill \end{array}\right],{\mathrm{R}}_{{\left(2\to 3\right)}^{\hbox{'}\hbox{'}\hbox{'}}}=\left[\begin{array}{c}\hfill \left\{2\kern1em \left( \mod \kern0.5em 11\right)\right\}/10\hfill \\ {}\hfill \left\{3\kern1.12em \left( \mod \kern0.5em 7\right)\right\}/6\hfill \\ {}\hfill 21.8\kern0.5em \left(\mathrm{mg}/\mathrm{dL}\right)\kern1em \hfill \\ {}\hfill 0.4\kern0.5em \left(1/24\mathrm{hrs}\right)\hfill \end{array}\right]. $$

These practical examples confirm the following relationship:

69 $$ {{\mathrm{R}}^3}^{\hbox{'}\hbox{'}\hbox{'}}=\left[{}_3^{\mathrm{N}}\mathrm{D}\left({\mathrm{g}}_3\right)\right]\left[{}_3^{\mathrm{N}}\mathrm{D}\left({\mathrm{g}}_2\right)\right]\left[{}_3^{\mathrm{N}}\mathrm{D}\left({\mathrm{g}}_1\right)\right]{\mathrm{R}}^3. $$

In general, with reference to (9), (12) and (25), we arbitrarily select two elements g_a_ and g_b_ (∈ GL(V^m^)), which satisfy R^m^ = R^ma^ and R^m^ = R^mb^, respectively. We also consider two perpendicular matrices $\left[{}_3^{\mathrm{N}}\mathrm{D}\left({\mathrm{g}}_{\mathrm{a}}\right)\right]$ and $\left[{}_3^{\mathrm{N}}\mathrm{D}\left({\mathrm{g}}_{\mathrm{b}}\right)\right]$. Then, for g_b_g_a_ (∈ GL(V^m^)), we confirm the following relationship:

70 $$ \left[{}_{\mathrm{m}}^{\mathrm{N}}\mathrm{D}\left({\mathrm{g}}_{\mathrm{b}}{\mathrm{g}}_{\mathrm{a}}\right)\right]=\left[{}_{\mathrm{m}}^{\mathrm{N}}\mathrm{D}\left({\mathrm{g}}_{\mathrm{b}}\right)\right]\left[{}_{\mathrm{m}}^{\mathrm{N}}\mathrm{D}\left({\mathrm{g}}_{\mathrm{a}}\right)\right]. $$

By (12) and (13), this expression satisfies the multiplication (product) requirement of a representation matrix. Naturally, all optional symbol ◊s presented in this section could be omitted, as in (31).

## Results

Above, we demonstrated that a group matrix representation of our proposed model can be constructed from N-tuple perpendicularly stratified vectors. In this construct, operators that correspond to the operators of patients’ diseased states are embedded in the individual matrix elements in each layer. Similarly, arbitrarily sized stratified vectors, denoting the number of patient disease states, are combined as column vectors into a square matrix. Additionally, the changes in the disease states can be expressed as plural products of the matrices.

## Discussion

In the *Model Assumptions* section, we mentioned that our concept might assist the treatment of clinical disease states, and described the stratified form of the vectors (matrices). The advantage of this notation is that vectors containing different operational unit (rules) are separately applied to individual R_j_s or [D(R_j_)]s. However, vectors composed of various operations cannot be treated in our model [19]. By not separating the individual components, we avoid interactions among respective components with different operational units (rules). In the conventional definition of vectors, the components are equally placed in the same plane, and mixtures of operational rules are considered to violate the rules of matrix operation. Specifically, the plural operational units cannot be defined in a unique square matrix because all manipulated rows and columns include a complicated expression, causing confusion as mentioned above (#). The concept of placing the individual components in a virtual direction enables independent manipulation of the individual components in a given layer. Through this device, we can define the matrix treatment and represent groups or rings through vectors composed of asymmetrically defined operational units (the products of non-mod/modulo arithmetic). We aimed for a group matrix representation in which addition or multiplication could be described by the same operator in each of the stratified layers (e.g., modulo-p_1_ addition). Briefly, the operations performed in respective layers are merely combined and ordered as vectors oriented vertically to the ordinal plane of the paper. We conjecture that this approach will enable an abstract treatment or data storing method, which could be formulated in future investigations. At least, such a formulation could improve the mathematical rigor of clinical medicine.

In our previous report [19], we defined the collective binary operation ◊ as a (non-modulo/modulo) arithmetic. In binary operations of R_j_s (between components such as r_(j)ν_◊r_(k)ν_), the extremes of ◊ are addition and multiplication. In such cases, ◊ might be a redundant operation because all of the manipulations can be performed by multiplying two elements, such as g_1_g_2_ or R_j_R_k_. Practical binary operations exclude simple addition between two matrices (g_1_ + g_2_ or R_j_ + R_k_), and the symbol ◊ or equivalent is not defined in any conventional algebra textbook. For example, from (8), (11) and (12), for a general linear group GL(V) with *n* dimensions and GL(V) = {f ∈ End_K_(V)|f^-1^ ∈ End_K_(V)}, we might also have GL(V) = GL(*n*, K) = {A∈ Mat_K_(*n*)| detA ≠ 0}. Whereas multiplication is closed when det(AB) = det(A)det(B), addition is not closed when det $$ \left(\left[\begin{array}{cc}\hfill 1\hfill & \hfill 0\hfill \\ {}\hfill 0\hfill & \hfill 1\hfill \end{array}\right]+\left[\begin{array}{cc}\hfill 0\hfill & \hfill 1\hfill \\ {}\hfill 1\hfill & \hfill 0\hfill \end{array}\right]\right)=0, $$ which contradicts detA ≠ 0 [23].

Thus, preceding a matrix manipulation, the symbol ◊ should be reinterpreted and replaced by an explicit operation (+, −, ×, /; non-mod or modular). Multiplications such as g_1_g_2_ should be specifically defined as the effects of medical operations or other clinical interventions, as illustrated in section 3.

In this paper (see Section 1), a composite of two elements of a set M is denoted R_j_◊R_k_, where ◊ is a modulo-p or non-modulo arithmetic. The symbol ◊ is included in the elements R_j_ (e.g. (4)). Strictly, to preserve the matrix operation postulates, the ◊ operator is applicable only to one-dimensional square matrices and *n*-dimensional diagonal matrices under the condition ‘◊ = ×’. Note that when *n* = 1, [A][B] = [R_(j)ν_][R_(k)ν_] = [R_(j)ν_][◊R_(k)ν_] = [r_(j)ν_][◊r_(k)ν_] = r_(j)ν_◊r_(k)ν_ = r_(j)ν_ × r_(k)ν_ = r_(j)ν_r_(k)ν_. In such cases, binary operations such as R_j_◊R_k_ are consistent with the product (multiplication) of two matrices. In other words, the notation r_(j)ν_◊r_(k)ν_ (where ◊ expresses the addition operator) is best avoided, and multiplication of two elements should be expressed in the conventional form r_(j)ν_r_(k)ν_. In the product of two multidimensional square matrices [A][B] (the ordinal multiplication of matrix A and B), the case ‘r_(j)ν_◊r_(k)ν_ = r_(j)ν_ + _(k)ν_’ in [A][B] = [R_(j)ν_][R_(k)ν_] = [r_(j)ν_][◊r_(k)ν_] is incompatible with the formal matrix definition. Therefore, in the present model, we might need to discard the ◊ operator or replace all ◊s by the desired manipulation (+ or ×) before commencing the matrix-treatment. If ◊ is addition, we simply add the matrices, and if ◊ is multiplication/division, we assume the ordinal multiplication (products) of matrices; for example, in the case of the multiplication operator non-modulo/modulo-p division, we perform multiplication by 1/R_j_, and modulo-p division by 1/R_j_ (mod p) = R_j_^−1^ (mod p).

One potential advantage of our model is that it orders the set M. Whether |M| is finite or infinite, the closure law of the modular approach prevents the dataset from exploding. We believe that under an appropriate investigation, our model might optimize data storage.

Our previous model [19] is not always amenable to matrix treatment, but suggests a vector approach for recording patient data of symptoms/clinical examinations. The operator ◊ confers no benefit in a vector-like representation. However, when expressing vector products of R_j_s in matrix form, we believe that ◊ is a useful binary operator for recording or storing datasets.

In this article, we proposed a double-meaning optional notation ◊ in (28), (53)–(56). This representation is not only simple (for example, R_1_ (=R_(0→1)_)) but the operator components can be linearly combined between states R_(j-1→j)_ and R_(j − 1 → j)'_ (in general, *j* = 1, 2,…, *m*). Thus, Eqs. (28), (53)–(56) can be interpreted in two ways; 1) as a series of changes of disease states in the perpendicular stratified vectors and 2) as linear combinations of states within the same layers, obtained in different sessions or under different conditions (e.g., with/without an intervention T). In fact, almost all bodily phenomena are nonlinear, but most medical data are interpreted in the linear formalism. If a dataset is acquired under ideal conditions such as within a short time interval, and the matrix is small (e.g., *m* = 2 or 3), the elements $\left[{}_{\mathrm{m}}^{\mathrm{N}}\mathrm{D}\left(\mathrm{g}\right)\right]$ in (28) and 29) might be determinable from statistical analyses. In this case, pre-, mid- and post-treatments might influence the elements $\left[{}_{\mathrm{m}}^{\mathrm{N}}\mathrm{D}\left(\mathrm{g}\right)\right]$, and the clinical course could be operationally expressed through (1), (21), (58), (59) or (62), with or without the ◊ symbols (non-mod/mod four arithmetic).

Regarding the sizes of the representation matrices, the one-dimensional square matrix $\left[{\mathrm{R}}_{\mathrm{j}}\right]\left(=,\left[{}_1^{\mathrm{N}}\mathrm{D}\left(\mathrm{g}\right)\right]\right)$is the simplest case that can be manipulated by Abelian operations such as R_j_R_k_ = R_k_R_j_. Conversely, plural-dimensional square matrices $\left[{}_{\mathrm{m}}^{\mathrm{N}}\mathrm{D}\left(\mathrm{g}\right)\right]$ (*m* ≥ 2) correspond to the non-Abelian (non-commutative) procedures in more complex phenomena [22]. Almost all medical phenomena might obey non-commutative laws. Moreover, many of these phenomena might be irreversible and not expressible as one-dimensional square matrices. The plural-dimensional representation matrix $\left[{}_{\mathrm{m}}^{\mathrm{N}}\mathrm{D}\left(\mathrm{g}\right)\right]$ (*m* ≥ 2) can be decomposed as the direct sum of smaller matrices. Therefore, rigorous investigations of $\left[{}_{\mathrm{m}}^{\mathrm{N}}\mathrm{D}\left(\mathrm{g}\right)\right]$ (*m* ≥ 2) are desired when the simpler representation is difficult or impossible in principle. From this viewpoint, we can regard [R_j_] in the one-dimensional square matrix in (17) and (18) as a mapping τ (with *m* = 1) in (20) and (23). Therefore, Eqs. (16) and (17) can be viewed as special cases of (41) or (42) (with *m* = 1), and (43) or (44) (with *m* = 1 and *n* = 1). Naturally, Eq. (43) could be viewed as a cross-section of (44) in the *ν*-th layer. In addition, (39) is a special case of (43) with *n* = 1, and (6) is a special case of (40) with *m* = 1.

In Eq. (29), we consider that operations with mixed rules are difficult to link in the conventional matrix representation. More generally, given a perpendicularly stratified vector R_j_ and appropriately postulated devices, an N-tuple combination among arbitrary r_(j)ν_s (where *v* is the component (layer) of R_j_; *v* = 1, 2,…, N) might be regarded as a tensor product r_(j)1_ ⊗ r_(j)2_ ⊗ … ⊗ r_(j)ν_ ⊗ … r_(j)(N − 1)_ ⊗ r_(j)N_ [9, 20–22]. By expressing the basis of perpendicularly stratified vectors (matrices) in terms of Φ_(N)_, we offer a generalized treatment of vectors such as (3), which might be applicable to other topics.

Our treatment also allows perpendicular stratified vectors (matrices). We conjecture that an ordinal treatment of these vectors is possible in respective layers. Therefore, the eigenvectors can be stratified, and the eigenvalues might be combinations of the highest N-tuple real numbers.

The limitations of the study should be noted. First, in the matrix representation, the concept of reducibility/irreducibility, more generally known as decomposability/non-decomposability, plays an important role. However, in our description, Abelian operations require only one-dimensional irreducible square matrices (i.e. scalars) [20–22]. Irreversible phenomena must be treated by non-Abelian operations. Crucially, a departure from the Abelian form implies a complex phenomenon in a medical procedure, which cannot be expressed as the direct sum of one-dimensional matrices. That is, the Abelian form is not applicable to complex phenomena.

Regarding the first limitation, there is no assurance that an entire medical procedure can be decomposed into the linear forms (29), (50) and (52). For instance, if a medical state is irreversible, it cannot be represented by Abelian operations. Especially, the elements of ${}_{\mathrm{m}}^{\mathrm{N}}\mathrm{D}$ in larger matrices (e.g., *m* ≥ 3) may not be determinable when the underlying phenomena are complex and nonlinear.

Second, the advantage of composite multiplications R_j_R_k_ seems unclear. Multiplication (including division) is necessitated by the ratio scale, which contains 0 and permits all four arithmetic operations [17]. In present research, multiplication (including division) plays a small role in clinical data analysis, being confined to the ratio between two data (e.g., relative risk, and the odds ratio of efficacy with/without a certain drug). However, the ratio scale is considered one of the ideal measures [17], and an arithmetic capability should be extendible to other fields of natural science such as biology, chemistry and physics, which often involve a conserved quantity such as energy and momentum. Therefore, we expect that formalizing the ratio scale would confer great advantages in future data analysis.

Third is the practical meaning of the expressions (29) (50) and (52). When a medical state is expressed as a plural-dimensional matrix (29) with simple linear combinations, the algebraic formalism is not readily linked to those of other natural sciences. Conversely, the simple expressions (17) cannot describe complex human phenomena. The direction in which the proposed formalism should be developed is ambiguous at present, and also requires investigation in future study.

Similarly, there is no assurance that a series of multiplications among plural representation matrices such as (33), (34), (39)−(44) can clearly detect a medical phenomenon. Moreover, the abstract formulation might not determine the mechanism of interest. From another perspective, if the dataset were acquired at an inappropriate time or state, the matrices would contain meaningless elements that would invalidate the analysis. Therefore, whether sufficient and valuable conditions are met needs to be determined. Additionally, the abstract formalism should clarify the mechanisms of actual medical states. Unfortunately, the present article has not progressed to this stage.

Finally, numbers or datasets could be duplicated in (20) and (23). Groups and related concepts preserve the order of a set, regardless of whether the set is finite or infinite. If the data are naively stored as (20) and (23), the data volume might explode, and the advantages of the stratified vector (matrix) formulation would be lost. Therefore, we require a methodology for reducing the data volume, possibly by exploiting the decomposition or reducibility properties.

Without doubt, our model is preliminary at this stage. Moreover, its practical applicability remains undeveloped. Although a rigorous mathematical treatment is beyond our expertise, we hope that this embryonic concept will be developed by professional mathematicians and physicists into a more descriptive representation of clinical medicine in future.

## Conclusions

Despite the brevity and incompleteness of our methodology, we consider that the stratified vector (matrix) representation of clinical data will lead to a more sophisticated formulation of clinical medicine in future.
